# Impact of deep or ovarian endometriosis on pelvic pain and quality of life: prospective cross‐sectional ultrasound study

**DOI:** 10.1002/uog.29150

**Published:** 2025-01-14

**Authors:** P. Chaggar, T. Tellum, N. Thanatsis, L. V. De Braud, T. Setty, D. Jurkovic

**Affiliations:** ^1^ EGA Institute for Women's Health, Faculty of Population Health Sciences University College London Hospital London UK; ^2^ Department of Gynaecology Oslo University Hospital Oslo Norway

**Keywords:** endometriosis, pelvic pain, quality of life, ultrasound

## Abstract

**Objective:**

To assess whether premenopausal women diagnosed with deep or ovarian endometriosis on transvaginal sonography (TVS) were more likely to suffer from dyspareunia and pelvic pain symptoms, and have a lower quality of life, compared to women without sonographically diagnosed deep or ovarian endometriosis.

**Methods:**

This was a prospective, cross‐sectional study carried out between February 2019 and October 2020 at the general gynecology clinic at University College London Hospital, London, UK. All premenopausal women aged 18–50 years, who were examined consecutively by a single experienced examiner and underwent a detailed TVS scan, were eligible for inclusion. Pregnant women and those who had received a previous diagnosis of endometriosis or who had experienced a hysterectomy or unilateral/bilateral oophorectomy were excluded. Sonographic findings consistent with deep or ovarian endometriosis were noted. All women completed the British Society of Gynaecological Endoscopy pelvic pain questionnaire. The primary outcome was to determine whether women with sonographic evidence of endometriosis were more likely to experience moderate‐to‐severe levels of dyspareunia (score of ≥ 4 on an 11‐point numerical rating scale (NRS)). Secondary outcomes included assessing moderate‐to‐severe levels of other pelvic pain symptoms (NRS score of ≥ 4), bowel symptoms (score of ≥ 2 on a 5‐point Likert scale) and quality of life, which was measured using the EuroQol‐5D‐3L (EQ‐5D) questionnaire. The number of women with pain scores ≥ 4 and bowel scores ≥ 2, as well as the mean EQ‐5D scores, were compared between the group with and that without sonographic evidence of endometriosis using logistic regression analysis, and multivariable analysis was used to adjust for demographic and clinical variables.

**Results:**

A total of 514 women were included in the final study population, of whom 146 (28.4%) were diagnosed with deep or ovarian endometriosis on TVS. On multivariable analysis, the presence of moderate‐to‐severe dyspareunia was not found to be associated with endometriosis. Moderate‐to‐severe dyspareunia was significantly associated with lower age (odds ratio (OR), 0.70 (95% CI, 0.56–0.89); *P* = 0.003) and a history of migraine (OR, 3.52 (95% CI, 1.42–8.77); *P* = 0.007), and it occurred significantly less frequently in women with non‐endometriotic ovarian cysts (OR, 0.47 (95% CI, 0.28–0.78); *P* = 0.003). There was also a trend towards a positive association between anxiety/depression and moderate‐to‐severe dyspareunia (OR, 1.94 (95% CI, 0.93–4.03); *P* = 0.08). Following multivariable analysis, the only symptoms that were significantly more common in women with endometriosis compared to those without were menstrual dyschezia (OR, 2.44 (95% CI, 1.59–3.78); *P* < 0.001) and difficulty emptying the bladder (OR, 2.56 (95% CI, 1.52–4.31); *P* < 0.001). Although not reaching statistical significance on multivariable analysis, dysmenorrhea (OR, 1.72 (95% CI, 0.92–3.20); *P* = 0.09) and lower EQ‐5D score (mean ± SD, 0.67 ± 0.33 *vs* 0.72 ± 0.28; *P* = 0.06) also occurred more frequently in women with sonographic evidence of endometriosis.

**Conclusions:**

The majority of pelvic pain symptoms did not differ significantly between women with and those without sonographic evidence of endometriosis, indicating that endometriosis may not always be the source of pelvic pain, even if present. This highlights the need to rule out other causes of pain in symptomatic endometriosis patients before considering surgical procedures, and to provide appropriate patient counseling. © 2024 The Author(s). *Ultrasound in Obstetrics & Gynecology* published by John Wiley & Sons Ltd on behalf of International Society of Ultrasound in Obstetrics and Gynecology.

## INTRODUCTION

Endometriosis is a recognized cause of chronic pelvic pain and reduced quality of life in women of reproductive age^1^, ^2^. However, the intricacies of the relationship between endometriosis and pelvic pain are poorly understood^3^, ^4^. This is a difficult relationship to investigate, as numerous factors can influence the degree of pain suffered by an individual, including emotional state, concomitant medical issues, expectations and attitudes regarding pain, mindset and exposure to exogenous substances^5^. Furthermore, endometriosis commonly co‐exists with other pelvic pathology, such as adenomyosis^6^, ^7^, ^8^ or fibroids^7^, and non‐gynecological conditions including fibromyalgia^9^ and irritable bowel syndrome^10^, all of which can contribute to pelvic pain.

Some studies have reported a positive correlation between endometriosis and dysmenorrhoea^11^, ^12^, chronic pelvic pain (CPP)^11^ and dyspareunia^11^, ^13^, ^14^, whereas others did not observe an association^12^, ^13^, ^15^. However, all these studies only included women undergoing surgery, which is likely to introduce selection bias, and they did not adjust for other factors that could influence pain.

Existing studies have not examined directly the relationship between the presence of endometriosis and bowel or urinary symptoms or quality of life, which are also thought to be affected by endometriosis^3^, ^16^.

It is important to improve our understanding of which pelvic pain symptoms are more likely to be associated with endometriosis, to help decipher whether endometriosis is the true cause of pain. This will be useful in improving patient selection for surgery. To do this accurately, women being managed expectantly or medically also need to be included. Transvaginal sonography (TVS) allows for investigation in women who do not require surgery and is now deemed to be comparable to laparoscopy for the diagnosis of endometriosis^3^. A recent study used TVS to investigate the relationship between pelvic pain symptoms and endometriosis^17^, however, rather than comparing women with and without endometriosis, it focused on the location of the disease.

The aim of this study was to determine whether premenopausal women attending our general gynecology clinic who were diagnosed sonographically with deep or ovarian endometriosis, were more likely to experience various pelvic pain symptoms and a reduced quality of life compared to women without deep or ovarian endometriosis.

## METHODS

### Study setting and patient population

This was a prospective, cross‐sectional study of women attending the general gynecology clinic at University College London Hospital, London, UK, between February 2019 and October 2020. Consecutive women who were seen by a single examiner, able to tolerate TVS and aged between 18 and 50 years, met the inclusion criteria. Exclusion criteria were a history of endometriosis (diagnosed by ultrasound, magnetic resonance imaging or laparoscopy), hysterectomy, unilateral/bilateral oophorectomy, current pregnancy or postmenopausal status (characterized by amenorrhea for ≥ 12 months, unrelated to exogenous hormones, breastfeeding or endocrine conditions). The ultrasound examiner (P.C.) had extensive experience in gynecological ultrasound (European Federation of Societies for Ultrasound in Medicine and Biology Level II)^18^, including in the diagnosis of endometriosis in a tertiary endometriosis center. Ethical approval was granted by The Liverpool Central Research Ethics Committee (Reference no. 19/NW/0050). The study was registered on Research Registry (no. researchregistry4828).

### Primary and secondary outcomes

The primary outcome of this study was to determine whether women with sonographic evidence of deep or ovarian endometriosis were more likely to experience moderate‐to‐severe dyspareunia than were women without sonographic evidence of deep or ovarian endometriosis.

Secondary outcomes were to determine whether women with sonographic findings consistent with deep or ovarian endometriosis were more likely to experience moderate‐to‐severe levels of other pelvic pain symptoms (premenstrual, menstrual, non‐cyclical and lower back pain), bladder symptoms (bladder pain, difficulty emptying the bladder) and bowel symptoms (menstrual and non‐menstrual dyschezia, frequent and urgent bowel movements, sensation of incomplete bowel emptying, constipation, menstrual hematochezia) and lower quality of life, compared to women without sonographic evidence of deep or ovarian endometriosis.

Various demographic and clinical variables and both gynecological and non‐gynecological diagnoses were assessed individually for their effect on the above‐mentioned pain and quality‐of‐life scores.

### Data collection and image acquisition

Demographic and clinical details were collected through an initial consultation and recorded in a secure hospital database (Viewpoint Bildverabeitung GmbH, Munich, Germany). Demographic information included age, body mass index (which was calculated using a calibrated scale and stadiometer in our clinic), ethnicity and smoking status. Clinical data collected consisted of presenting complaint, menstrual history, obstetric history (gravidity, parity, vaginal births comprising spontaneous and instrumental deliveries, Cesarean sections) and other conditions commonly associated with pelvic pain and reduced quality of life (anxiety and depression, chronic pain syndrome, chronic fatigue syndrome, fibromyalgia, irritable bowel disease, irritable bowel syndrome, migraine). Menstrual history data collected included regularity of menstrual bleeding (regular: shortest‐to‐longest cycle variation of up to 7–9 days, depending on age; irregular: shortest‐to‐longest cycle variation exceeding 8–10 days, depending on age), frequency of menstrual bleeding (absent: amenorrhea; infrequent: > 38 days; normal: 24–38 days; frequent: < 24 days; or variable) and duration of menstrual bleeding (normal: ≤ 8 days; prolonged: > 8 days; or variable)^19^.

Once patients had given formal written consent for participation in the study, they reported their clinical symptoms using the standardized British Society of Gynaecological Endoscopy pelvic pain questionnaire^16^. The severity of pelvic pain symptoms was rated on an 11‐point numerical rating scale, comprising premenstrual and menstrual pain, non‐cyclical pelvic pain, dyspareunia, menstrual and non‐menstrual dyschezia, lower back pain and bladder symptoms including bladder pain and difficulty emptying the bladder. A score of ≥ 4 was used to define moderate‐to‐severe pain^20^. Bowel symptoms (frequency and urgency of bowel movements, sensation of incomplete emptying, constipation and menstrual hematochezia) were graded on a 5‐point Likert scale^20^. A score of ≥ 2 was used to define moderate‐to‐severe bowel symptoms^20^. Dichotomous data were collected regarding the use of analgesia or hormonal treatment (yes or no) and length of time trying to conceive (< 18 months or >18 months). Health‐related quality of life was assessed using the validated EuroQol‐5D‐3L (EQ‐5D) questionnaire^21^. This comprises two components, one of which evaluates mobility, self‐care, daily activities, pain and discomfort, and anxiety and depression in five questions. An EQ‐5D index score is calculated using the responses from these questions, where 1 represents perfect health and 0 represents a health state equivalent to death. The second component consists of a 100‐point visual analog scale, referred to as the EQ Visual Analog Scale (EQ‐VAS), where women rate their overall health status, with higher scores denoting better health.

All patients underwent TVS using a 7.5‐MHz probe (Voluson E8, GE Medical Systems, Zipf, Austria). For assessment of endometriosis, the standardized approach described by the International Deep Endometriosis Analysis group was employed^22^. The features used to identify endometriomas, endometriotic nodules and pouch of Douglas obliteration have been described in detail in a previous study, as have the methods used to measure the lesions^8^.

Other gynecological abnormalities were diagnosed in line with criteria outlined in the international Morphological Uterus Sonographic Assessment group consensus statement^23^ for adenomyosis and fibroids, International Endometrial Tumor Analysis group consensus statement^24^ and more recent literature^25^ for cervical and endometrial polyps, the revised American Society for Reproductive Medicine classification for congenital uterine anomalies^26^, recent literature for accessory cavitated uterine malformation^27^ and dilated pelvic veins^28^ and a described pattern recognition model for non‐endometriotic ovarian cysts^29^.

Transabdominal ultrasound of the kidneys was also performed routinely using a 3.5‐MHz probe (Voluson E8) to check for hydronephrosis and other abnormalities, including renal cysts.

### Sample size calculation and statistical analysis

Dyspareunia is a symptom that appears to be more consistently associated with endometriosis compared with other pelvic pain symptoms, demonstrated in several studies as summarized by Vercellini *et al*.^30^ as well as in studies published subsequently^31^, ^32^. It could also be argued that dyspareunia is less likely to be caused by concomitant pelvic pathology compared with dysmenorrhea and other pelvic pain symptoms linked to endometriosis, such as CPP. For this reason, the primary outcome and consequently the sample‐size calculation were based on this symptom. It has been reported that 30% of women with endometriosis experience dyspareunia, compared to 13% of women without endometriosis^33^. Sample‐size calculation demonstrated that 182 women were required to show this difference, with 91 women in each group, a power of 80% and a confidence interval of 95%.

Stata version 15.1 (StataCorp LLC, TX, USA) was used for statistical analysis. Normally distributed continuous data are expressed as mean ± SD and non‐normally distributed data as median (interquartile range). The distribution of data was ascertained by examination of skewness and kurtosis. Categorical data are presented as percentages with 95% CI or as *n* (%). The chi‐square test was used to compare categorical variables between patients with and those without endometriosis, and the Fisher's exact test was used when the sample size was small. Continuous variables were compared using the unpaired *t*‐test for normally distributed variables or the Mann–Whitney *U*‐test for non‐normally distributed variables.

Logistic regression analysis was used to calculate whether the presence of various demographic and clinical factors influenced pain and quality‐of‐life scores, as well as responses to questions regarding fertility. This was an explorative analysis. After adjusting for the statistically significant predictor variables, multivariable analysis was performed to determine whether any initial differences seen in the outcome variables between the groups with and without deep or ovarian endometriosis remained. A backwards selection procedure was then used to retain only the statistically significant variables in the final multivariable analysis model. Odds ratios were used to quantify these differences. *P* < 0.05 was considered to indicate statistical significance.

## RESULTS

**Figure 1:**
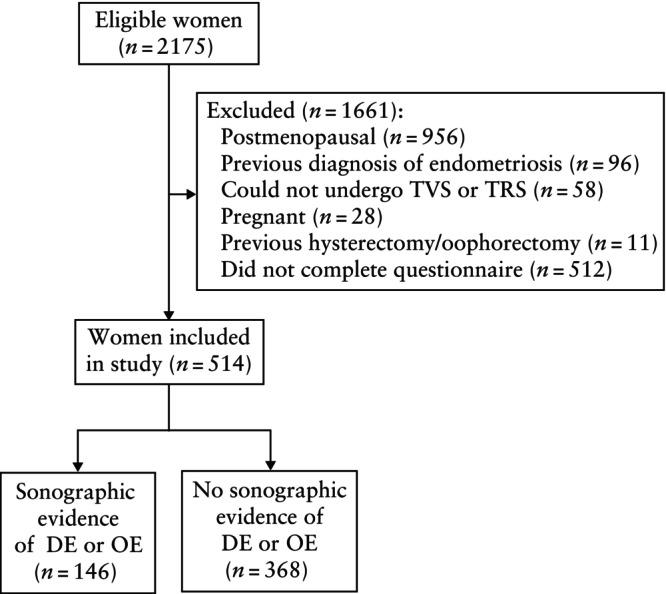
Flowchart summarizing inclusion of patients in study population. DE, deep endometriosis; OE, ovarian endometriosis; TRS, transrectal sonography; TVS, transvaginal sonography.

During the 20‐month study period, 2175 eligible women were identified, of whom 514 were included in the final study sample. A flowchart summarizing the study population is shown in Figure 1.

The primary indications for attendance in the groups of women with (146/514 (28.4%)) and those without (368/514 (71.6%)) sonographic findings consistent with deep or ovarian endometriosis are shown in Table 1. The most common reasons for referral were abnormal uterine bleeding (182/514 (35.4%)) and CPP (125/514 (24.3%)).

**Table 1:** Primary indication for attendance at general gynecology clinic, according to presence or absence of endometriosis on transvaginal ultrasound (*n* = 514)

Indication	Endometriosis present (*n* = 146)	Endometriosis absent (*n* = 368)	*P*
Abnormal uterine bleeding	52 (35.6 (27.9–44.0))	130 (35.3 (30.4–40.5))	0.95
Heavy uterine bleeding and dysmenorrhea	19 (13.0 (8.0–19.6))	30 (8.2 (5.6–11.4))	0.91
Heavy uterine bleeding only	18 (12.3 (7.5–18.8))	58 (15.8 (12.2–19.9))	0.32
Intermenstrual bleeding	11 (7.5 (3.8–13.1))	28 (7.6 (5.1–10.8))	0.98
Irregular menstrual bleeding	4 (2.7 (0.8–6.9))	14 (3.8 (2.1–6.3))	0.55
Chronic pelvic pain	37 (25.3 (18.5–33.2))	88 (23.9 (19.6–28.6))	0.73
Dysmenorrhea only	28 (19.2 (13.1–26.5))	37 (10.1 (7.2–13.6))	0.005
Follow‐up of ovarian cyst	6 (4.1 (1.5–8.7))	23 (6.3 (4.0–9.2))	0.34
Infertility	4 (2.7 (0.8–6.9))	8 (2.2 (0.9–4.2))	0.75
Deep dyspareunia	2 (1.4 (0.2–4.9))	16 (4.3 (2.5–7.0))	0.98
Bloating	2 (1.4 (0.2–4.9))	9 (2.4 (1.1–4.6))	0.74
Postcoital bleeding	1 (0.7 (0.08–3.2))	11 (3.0 (1.5–5.3))	0.19
Contraception discussion	0 (0 (0.0–2.5))	11 (3.0 (1.5–5.3))	0.04
Other	14 (9.6 (5.3–15.6))	35 (9.5 (6.7–13.0))	0.98

Data are given as *n* (% (95% CI)).

Demographic characteristics and medical and reproductive history of the study participants are shown in Table 2. Variables were comparable in women with and those without sonographic evidence of endometriosis, except that women with endometriosis were more likely to have a history of infertility (*P* = 0.008) and migraine (*P* = 0.04), and less likely to suffer from anxiety or depression (*P* = 0.04).

**Table 2:** Demographic and clinical characteristics of 514 women included in study, according to presence or absence of endometriosis on transvaginal ultrasound

Variable	Endometriosis present	OR (95% CI)*	*P*
Age†	—	1.20 (0.94–1.53)	0.15
BMI category‡			0.21
Normal	64/234 (27.4)	1	
Underweight	3/18 (16.7)	0.53 (0.15–1.90)	
Pre‐obesity	35/100 (35.0)	1.43 (0.87–2.36)	
Obesity Class 1	10/50 (20.0)	0.66 (0.31–1.41)	
Obesity Class 2/3	12/36 (33.3)	1.33 (0.63–2.81)	
Ethnicity			0.97
White	78/275 (28.4)	1	
Black	18/61 (29.5)	1.06 (0.57–1.94)	
Asian	15/58 (25.9)	0.88 (0.46–1.68)	
Mixed/other	35/120 (29.2)	1.04 (0.65–1.67)	
Smoking status§			0.96
Non‐smoker	92/318 (28.9)	1	
Ex‐smoker	34/120 (28.3)	0.97 (0.61–1.55)	
Current smoker	20/73 (27.4)	0.93 (0.52–1.64)	
Regular periods¶			0.14
No	31/129 (24.0)	1	
Yes	111/358 (31.0)	1.42 (0.90–2.25)	
Period frequency			0.20
Normal	98/314 (31.2)	1	
Infrequent	4/24 (16.7)	0.44 (0.15–1.32)	
Frequent	4/15 (26.7)	0.80 (0.11–2.58)	
Variable	36/134 (26.9)	0.81 (0.47–1.27)	
No periods	4/27 (14.8)	0.38 (0.13–1.14)	
Period length¶			0.48
Normal	119/402 (29.6)	1	
Prolonged	8/22 (36.4)	1.36 (0.56–3.32)	
Variable	15/63 (23.8)	0.74 (0.40–1.38)	
Contraception method			0.17
None	103/334 (30.8)	1	
Hormonal	31/120 (25.8)	0.78 (0.49–1.25)	
Non‐hormonal	12/60 (20.0)	0.56 (0.29–1.10)	
Gravidity			0.13
0	78/255 (30.6)	1	
1	34/107 (31.8)	1.06 (0.65–1.72)	
≥ 2	34/152 (22.4)	0.65 (0.41–1.04)	
Parity			0.11
0	101/339 (29.8)	1	
1	23/69 (33.3)	1.18 (0.68–2.05)	
≥ 2	22/106 (20.8)	0.62 (0.37–1.04)	
Prior vaginal delivery**			0.44
0	11/45 (24.4)	1	
1	17/53 (32.1)	1.45 (0.60–3.56)	
≥ 2	17/77 (22.1)	0.88 (0.37–2.08)	
Prior Cesarean delivery**			0.24
0	34/115 (29.6)	1	
1	6/36 (16.7)	0.48 (0.18–1.25)	
≥ 2	5/24 (20.8)	0.63 (0.22–1.82)	
Spontaneous miscarriage††			0.53
0	47/185 (25.4)	1	
1	16/50 (32.0)	1.38 (0.70–2.73)	
≥ 2	5/24 (20.8)	0.77 (0.27–2.18)	
SMM††			0.26
0	60/237 (25.3)	1	
≥ 1	8/22 (36.4)	1.698 (0.67–4.22)	
Prior ectopic pregnancy††			0.71
0	64/246 (26.0)	1	
≥ 1	4/13 (30.8)	1.26 (0.38–4.25)	
MTOP††			0.41
0	62/229 (27.1)	1	
≥ 1	6/30 (20.0)	0.67 (0.26–1.73)	
STOP††			0.40
0	53/192 (27.6)	1	
≥ 1	15/67 (22.4)	0.76 (0.39–1.46)	
History of infertility			0.008
No	114/436 (26.1)	1	
Yes	32/78 (41.0)	1.96 (1.19–3.24)	
Irritable bowel syndrome			0.06
No	144/492 (29.3)	1	
Yes	2/22 (9.1)	0.24 (0.06–1.05)	
Inflammatory bowel disease			0.88
No	145/510 (28.4)	1	
Yes	1/4 (25.0)	0.84 (0.09–8.13)	
Anxiety/depression			0.04
No	142/481 (29.5)	1	
Yes	4/33 (12.1)	0.33 (0.11–0.95)	
Fibromyalgia			0.91
No	143/504 (28.4)	1	
Yes	3/10 (30.0)	1.08 (0.28–4.24)	
Chronic fatigue syndrome			0.51
No	145/512 (28.3)	1	
Yes	1/2 (50.0)	2.53 (0.16–40.7)	
Chronic pain syndrome			—
No	146/513 (28.5)	1	
Yes	0/1 (0)	‡‡	
Migraine			0.04
No	134/488 (27.5)	1	
Yes	12/26 (46.2)	2.26 (1.02–5.02)	
Asthma			0.91
No	133/467 (28.5)	1	
Yes	13/47 (27.7)	0.96 (0.49–1.88)	
Autoimmune disease			0.21
No	144/499 (28.9)	1	
Yes	2/15 (13.3)	0.38 (0.08–1.70)	
Hypothyroidism			0.07
No	133/484 (27.5)	1	
Yes	13/30 (43.3)	2.02 (0.95–4.27)	
Diabetes			0.91
No	143/504 (28.4)	1	
Yes	3/10 (30.0)	1.08 (0.28–4.24)	
Hypertension			0.45
No	144/503 (28.6)	1	
Yes	2/11 (18.2)	0.55 (0.12–2.60)	

Data are given as *n/N* (%), unless indicated otherwise.

* Calculated as odds of outcome in women with endometriosis relative to odds in women without endometriosis.

† Odds ratio (OR) given for 10‐year increase in age.

‡ *n* = 438 owing to missing data.

§ *n* = 511 owing to missing data.

¶ Analysis omitted amenorrheic women.

** Analysis excluded nulliparous women.

†† Analysis excluded nulligravid women.

‡‡ Unable to calculate OR as all women in one group had the same outcome.

BMI, body mass index; MTOP, medical termination of pregnancy; SMM, surgical management of miscarriage; STOP, surgical termination of pregnancy.

Pelvic abnormalities, inclusive of endometriosis, were detected in 425 (82.7%) of the women; 177 (34.4%) participants were diagnosed with a single abnormality, and 248 (48.2%) were found to have multiple abnormalities. In the endometriosis subgroup, concomitant pelvic abnormalities were found in 137/146 (93.8%) patients, of which pelvic adhesions (103/146 (70.5%)) were the most common (Table 3).

**Table 3:** Other ultrasound diagnoses of 514 women included in study, according to presence or absence of endometriosis on transvaginal ultrasound

Diagnosis/history	Endometriosis present*(n =* 146)	Endometriosis absent *(n =* 368)	OR (95% CI)*	*P*
Pelvic adhesions				< 0.001
Yes	103 (70.5)	40 (10.9)	1	
No	43 (29.5)	328 (89.1)	19.6 (12.1–31.9)	
Adenomyosis				< 0.001
Yes	64 (43.8)	86 (23.4)	1	
No	82 (56.2)	282 (76.6)	2.56 (1.70–3.84)	
Uterine fibroid(s)				0.18
Yes	55 (37.7)	116 (31.5)	1	
No	91 (62.3)	252 (68.5)	1.31 (0.88–1.96)	
Non‐endometriotic ovarian cyst(s)				0.66
Yes	23 (15.8)	64 (17.4)	1	
No	123 (84.2)	304 (82.6)	0.88 (0.53–1.49)	
Endometrial and/or cervical polyp(s)				0.47
Yes	17 (11.6)	35 (9.5)	1	
No	129 (88.4)	333 (90.5)	1.25 (0.68–2.32)	
Dilated pelvic vein(s)†				0.90
Yes	14/112 (12.5)	35/291 (12.0)	1	
No	98/112 (87.5)	256/291 (88.0)	1.04 (0.54–2.03)	
Polycystic ovarian morphology				0.10
Yes	12 (8.2)	50 (13.6)	1	
No	134 (91.8)	318 (86.4)	0.57 (0.29–1.10)	
Dilated Fallopian tube(s)				0.001
Yes	11 (7.5)	5 (1.4)	1	
No	135 (92.5)	363 (98.6)	5.92 (2.02–17.3)	
Congenital uterine anomaly				0.79
Yes	3 (2.1)	9 (2.4)	1	
No	143 (97.9)	359 (97.6)	0.84 (0.22–3.13)	
Renal abnormality				0.51
Yes	1 (0.7)	1 (0.3)	1	
No	145 (99.3)	367 (99.7)	2.53 (0.16–40.7)	
ACUM				—
Yes	0 (0)	2 (0.5)	1	
No	146 (100)	366 (99.5)	‡	

Data are given as *n* (%) or *n*/*N* (%), unless indicated otherwise.

* Calculated as odds of outcome in women with endometriosis relative to odds in women without endometriosis.

† *n* = 403 owing to missing data.

‡ Unable to calculate odds ratio (OR) as all women in one group had the same outcome.

ACUM, accessory cavitated uterine malformation.

On univariable analysis, premenstrual pain (*P* = 0.02), menstrual dyschezia (*P* < 0.001), bladder pain (*P* = 0.01), difficulty emptying the bladder (*P* < 0.001), incomplete emptying of the bowel (*P* = 0.04) and the use of progestogen (*P* = 0.04) and use of non‐steroidal anti‐inflammatory drugs (*P* < 0.001) occurred more frequently in women with sonographic evidence of endometriosis than it did in those without (Table 4). There was no significant difference between the two groups when comparing other pain and bowel symptoms, time trying to conceive or EQ‐VAS scores (Tables 4 and 5). On multivariable analysis, once confounding variables had been accounted for, only menstrual dyschezia (*P* < 0.001) and difficulty emptying the bladder (*P* < 0.001) remained statistically significant. Although not reaching statistical significance, there was a trend towards dysmenorrhea (*P* = 0.09) and lower EQ‐5D score (*P* = 0.06) occurring more commonly in women with sonographic evidence of endometriosis than in those without (Tables 4 and 5).

**Table 4:** Outcomes of 514 women included in study, according to presence or absence of endometriosis on transvaginal ultrasound

				*P*	
Outcome	Endometriosis present	Endometriosis absent	OR* (95% CI)	Univariable analysis	Multivariable analysis††
Moderate‐to‐severe pain symptoms					
Premenstrual pain†	107/142 (75.4)	219/342 (64.0)	1.72 (1.10–2.67)	0.02	—
Dysmenorrhea‡	124/142 (87.3)	274/343 (79.9)	1.73 (0.99–3.04)	0.05	0.09
Non‐cyclical pain	68/146 (46.6)	156/368 (42.4)	1.18 (0.81–1.74)	0.39	—
Dyspareunia	73/146 (50.0)	161/368 (43.8)	1.29 (0.88–1.89)	0.20	—
Menstrual dyschezia‡	68/142 (47.9)	97/343 (28.3)	2.33 (1.56–3.49)	< 0.001	< 0.001
Non‐menstrual dyschezia	37/146 (25.3)	74/367 (20.2)	1.34 (0.86–2.11)	0.20	—
Lower back pain	101/146 (69.2)	234/366 (63.9)	1.27 (0.84–1.91)	0.26	—
Bladder pain	38/146 (26.0)	60/368 (16.3)	1.81 (1.14–2.87)	0.01	—
Difficulty emptying bladder	34/146 (23.3)	39/368 (10.6)	2.56 (1.54–4.25)	< 0.001	< 0.001
Moderate‐to‐severe bowel symptoms					
Frequent movements	110/146 (75.3)	277/368 (75.3)	1.00 (0.64–1.57)	0.99	—
Urgent movements	55/146 (37.7)	120/368 (32.6)	1.25 (0.84–1.86)	0.28	—
Incomplete emptying	58/146 (39.7)	111/368 (30.2)	1.53 (1.02–2.27)	0.04	—
Constipation	63/146 (43.2)	130/368 (35.3)	1.39 (0.94–2.05)	0.10	—
Menstrual hematochezia§	22/142 (15.5)	34/345 (9.9)	1.68 (0.94–2.98)	0.08	—
Hormone use					
OCP	11/146 (7.5)	28/368 (7.6)	0.99 (0.48–2.04)	0.98	**
Mirena	15/146 (10.3)	27/368 (7.3)	1.45 (0.75–2.81)	0.28	**
GnRH analog	0/146 (0)	0/368 (0)	—	—	**
GnRH analog and estrogen	0/146 (0)	0/368 (0)	—	—	**
Progestogen	5/146 (3.4)	33/368 (9.0)	0.36 (0.14–0.94)	0.04	**
Aromatase inhibitor	0/146 (0)	0/368 (0)	—	—	**
HRT	0/146 (0)	0/368 (0)	—	—	**
Any hormone	31/146 (21.2)	88/368 (23.9)	0.86 (0.54–1.36)	0.52	**
Analgesia use					
Paracetamol	109/146 (74.7)	276/368 (75.0)	0.98 (0.63–1.53)	0.94	
NSAID	110/146 (75.3)	215/368 (58.4)	2.17 (1.42–3.34)	< 0.001	**
Opiate	24/146 (16.4)	48/368 (13.0)	1.31 (0.77–2.23)	0.32	**
Any analgesia	127/146 (87.0)	305/368 (82.9)	1.38 (0.79–2.40)	0.25	**
Trying to conceive for > 18 months¶	8/17 (47.1)	24/53 (45.3)	1.07 (0.36–3.21)	0.90	**

Data are given as *n*/*N* (%), unless indicated otherwise.

* Calculated as odds of outcome in women with endometriosis relative to odds in women without endometriosis.

† *n* = 484 owing to omission of amenorrheic women and exclusion of three women owing to missing data.

‡ *n* = 485 owing to omission of amenorrheic women and exclusion of two women owing to missing data.

§ *n* = 487 owing to omission of amenorrheic women.

¶ Analysis for subgroup of women trying to conceive only.

** Multivariable analysis not conducted for these variables.

†† *P*‐values presented only for variables that were significant/borderline significant on multivariable analysis using a backward selection procedure.

GnRH, gonadotropin‐releasing hormone; HRT, hormone replacement therapy; NSAID, non‐steroidal anti‐inflammatory drug; OCP, oral contraceptive pill; OR, odds ratio.

**Table 5:** Quality‐of‐life outcomes of 514 women included in study, according to presence or absence of endometriosis on transvaginal ultrasound

				*P*	
Outcome	Endometriosis present	Endometriosis absent	Regression coefficient (95% CI)	Univariable analysis	Multivariable analysis*
EQ‐5D score	0.67 ± 0.33	0.72 ± 0.28	−0.05 (−0.11 to 0.01)	0.09	0.06
EQ‐VAS score	67.1 ± 20.1	69.7 ± 19.9	−2.7 (−6.2 to 1.2)	0.17	—

Data are given as mean ± SD, unless indicated otherwise.

* *P*‐values presented only for variables that were significant/borderline significant on multivariable analysis using a backward selection procedure. EQ‐5D, EuroQol‐5D‐3L; EQ‐VAS, EuroQol Visual Analog Scale.

There were significantly more women with moderate‐ to‐severe menstrual dyschezia, dysmenorrhea and difficulty emptying the bladder who had sonographic evidence of posterior compartment deep endometriotic lesions, compared to those with no or mild symptoms (59/165 (35.8%) *vs* 63/320 (19.7%), *P* = 0.0001; 125/398 (31.4%) *vs* 17/87 (19.5%), *P* < 0.05; and 30/73 (41.1%) *vs* 96/441 (21.8%), *P* = 0.0004, respectively).

Following analysis of the effects of various demographic and clinical variables and pelvic pathologies diagnosed on TVS in the presence of moderate‐to‐severe dyspareunia, lower age (*P* = 0.001) and history of migraine (*P* = 0.006) were found to be positively associated with dyspareunia. However, moderate‐to‐severe dyspareunia was significantly less common in women with non‐endometriotic ovarian cysts (*P* = 0.003) compared to those without non‐endometriotic ovarian cysts (Table 6). These factors remained statistically significant after adjustment for possible confounding variables on multivariable analysis (*P* = 0.003, *P* = 0.007 and *P* = 0.003, respectively) (Table 7). Although not reaching statistical significance, there was a trend towards a positive association between anxiety/depression and moderate‐to‐severe dyspareunia (*P* = 0.08) (Table 7).

**Table 6:** Univariable analysis of association of demographic and clinical variables with moderate‐to‐severe dyspareunia

Variable	Dyspareunia	OR (95% CI)	*P*
Endometriosis			0.20
No	161/368 (43.8)	1	
Yes	73/146 (50.0)	1.29 (0.88–1.89)	
Age*	—	0.67 (0.54–0.85)	0.001
Ethnicity			0.15
White	122/275 (44.4)	1	
Black	29/61 (47.5)	1.14 (0.65–1.98)	
Asian	34/58 (58.6)	1.78 (1.00–3.15)	
Mixed/other	49/120 (40.8)	0.87 (0.56–1.34)	
BMI†	—	0.92 (0.68–1.24)	0.58
BMI category‡			0.74
Normal	106/234 (45.3)	1	
Underweight	9/18 (50.0)	1.21 (0.46–3.15)	
Pre‐obesity	46/100 (46.0)	1.03 (0.64–1.65)	
Obesity Class 1	18/50 (36.0)	0.68 (0.36–1.28)	
Obesity Class 2/3	15/36 (41.7)	0.86 (0.42–1.76)	
Smoking status§			0.39
Non‐smoker	138/318 (43.4)	1	
Ex‐smoker	56/120 (46.7)	1.14 (0.75–1.74)	
Current smoker	38/73 (52.1)	1.42 (0.85–2.36)	
Regular periods¶			0.20
No	64/129 (49.6)	1	
Yes	154/358 (43.0)	0.77 (0.51–1.15)	
Period frequency			0.69
Normal	139/314 (44.3)	1	
Infrequent	11/24 (45.8)	1.07 (0.46–2.45)	
Frequent	7/15 (46.7)	1.10 (0.39–3.11)	
Variable	61/134 (45.5)	1.05 (0.70–1.58)	
No periods	16/27 (59.3)	1.83 (0.82–4.07)	
Period length¶			0.34
Normal	186/402 (46.3)	1	
Prolonged	8/22 (36.4)	0.66 (0.27–1.62)	
Variable	24/63 (38.1)	0.71 (0.41–1.23)	
Gravidity			0.58
0	122/255 (47.8)	1	
1	46/107 (43.0)	0.82 (0.52–1.30)	
≥ 2	66/152 (43.4)	0.84 (0.56–1.25)	
Parity			0.63
0	157/339 (46.3)	1	
1	33/69 (47.8)	1.06 (0.63–1.78)	
≥ 2	44/106 (41.5)	0.82 (0.53–1.28)	
Prior Cesarean delivery**			0.44
0	49/115 (42.6)	1	
1	19/36 (52.8)	1.51 (0.71–3.19)	
≥ 2	9/24 (37.5)	0.81 (0.32–2.00)	
Prior vaginal delivery**			0.65
0	22/45 (48.9)	1	
1	21/53 (39.6)	0.69 (0.31–1.53)	
≥ 2	34/77 (44.2)	0.83 (0.40–1.73)	
Analgesia use			0.42
No	34/82 (41.5)	1	
Yes	200/432 (46.3)	1.22 (0.75–1.96)	
Hormonal contraception			0.05
No	170/394 (43.1)	1	
Yes	64/120 (53.3)	1.51 (1.00–2.27)	
Irritable bowel syndrome			0.20
No	221/492 (44.9)	1	
Yes	13/22 (59.1)	1.77 (0.74–4.22)	
Inflammatory bowel disease			0.86
No	232/510 (45.5)	1	
Yes	2/4 (50.0)	1.20 (0.17–8.57)	
Anxiety/depression			0.08
No	214/481 (44.5)	1	
Yes	20/33 (60.6)	1.92 (0.93–3.95)	
Fibromyalgia			0.36
No	228/504 (45.2)	1	
Yes	6/10 (60.0)	1.82 (0.51–6.51)	
Migraine			0.006
No	215/488 (44.1)	1	
Yes	19/26 (73.1)	3.45 (1.42–8.35)	
Adenomyosis			0.80
No	167/364 (45.9)	1	
Yes	67/150 (44.6)	0.95 (0.65–1.40)	
Uterine fibroid(s)			0.07
No	166/343 (48.4)	1	
Yes	68/171 (39.8)	0.70 (0.49–1.02)	
Non‐endometriotic ovarian cyst(s)			0.003
No	207/427 (48.5)	1	
Yes	27/87 (31.0)	0.48 (0.29–0.78)	
Pelvic adhesions			0.71
No	167/371 (45.0)	1	
Yes	67/143 (46.9)	1.08 (0.73–1.59)	
Dilated pelvic vein(s)††			0.30
No	160/354 (45.2)	1	
Yes	26/49 (53.1)	1.37 (0.75–2.49)	
Dilated Fallopian tube(s)			0.39
No	225/498 (45.2)	1	
Yes	9/16 (56.3)	1.56 (0.57–4.25)	

Data are given as *n*/*N* (%), unless indicated otherwise.

* Odds ratio (OR) given for 10‐year increase in age.

† OR given for 10‐kg/m^2^ increase in body mass index (BMI).

‡ *n* = 438 owing to missing data.

§ *n* = 511 owing to missing data.

¶ Women with no periods excluded (*n* = 487).

** Analysis excluded nulliparous women (*n* = 175).

†† *n* = 403 owing to missing data.

**Table 7:** Multivariable analysis of factors associated with moderate‐to‐severe dyspareunia

Variable	OR (95% CI)	*P*
Age*	0.70 (0.56–0.89)	0.003
Anxiety/depression		0.08
No	1	
Yes	1.94 (0.93–4.03)	
History of migraine		0.007
No	1	
Yes	3.52 (1.42–8.77)	
Non‐endometriotic ovarian cyst(s)		0.003
No	1	
Yes	0.47 (0.28–0.78)	

* Odds ratio (OR) given for 10‐year increase in age.

The results of the univariable and multivariable analyses assessing the effects of these variables on other pelvic pain, urinary and bowel symptoms and EQ‐5D scores are detailed in Tables S1–S29.

## DISCUSSION

Following adjustment for confounding factors, our study found no difference in the presence of moderate‐ to‐severe dyspareunia between women with and those without sonographic evidence of endometriosis. However, moderate‐to‐severe menstrual dyschezia and difficulty emptying the bladder were significantly more common in women with sonographic evidence of endometriosis, with dysmenorrhea and lower EQ‐5D score also occurring more commonly, but not reaching statistical significance. Lower age, a history of migraine and anxiety/depression were all associated (either significantly or with a trend towards significance) with moderate‐to‐severe dyspareunia.

Several previous studies have also investigated the relationship between the presence of endometriosis and dyspareunia^11^, ^12^, ^13^, ^14^, ^15^, but only two reported findings similar to ours^12^, ^15^. These appear to be the only five published studies that have also examined the association between the presence of endometriosis and other pain symptoms, namely menstrual pain and CPP. The trend towards moderate‐to‐severe dysmenorrhea being more common in women with endometriosis than in those without, which we identified in our study, was also reported in two studies in the literature^11^, ^12^, whereas other studies observed no association^13^, ^15^. However, not all studies used a standardized measure of pain^11^, ^15^ or confirmed endometriosis histologically^11^, ^13^.

It has been reported that the prevalence of endometriosis in asymptomatic women could be as high as 44%^34^. The inclusion of this subgroup of endometriosis patients in our study may have contributed to the discrepancy between our results and those of previous studies, which only included symptomatic women who were undergoing surgery. Additionally, ours was the only study to adjust for confounding variables.

The positive association between the presence of endometriosis confirmed on ultrasound and moderate‐to‐ severe menstrual dyschezia and dysmenorrhea could be due to significantly more of these women having posterior compartment deep endometriotic lesions, compared to those with no or mild symptoms. Previous studies have also shown a similar association with menstrual dyschezia^32^, ^35^, ^36^ and dysmenorrhea^36^, ^37^.

The relationship between difficulty emptying the bladder and location of endometriosis has not been previously investigated. However, some studies have described a positive anatomical association between bladder pain and deep anterior compartment endometriosis^12^, ^32^, ^35^. Our study did not find this association, probably owing to the small sample size of women with deep anterior compartment endometriosis. Interestingly, women who reported moderate‐to‐severe difficulty emptying the bladder were more likely to have sonographic findings consistent with posterior compartment deep endometriosis, compared to those with no or mild symptoms.

The association between particular symptoms and deep endometriotic lesions in the posterior compartment could be explained by nerve involvement^38^. Infiltration of the sacral plexus by deep endometriotic nodules could cause symptoms including menstrual dyschezia^39^, ^40^ and symptoms unrelated to anatomical location, including difficulty emptying the bladder. The sacral plexus comprises the pudendal nerve, which supplies areas including the perineum, vagina, anal canal and urethra. This could also explain the discrepancies between different studies, as the size, depth and precise location of the nodules is likely to influence which lesions affect such nerves.

In our study, all menstruation‐specific symptoms, except menstrual hematochezia, were positively associated with endometriosis, indicating that they may be valuable markers for its presence. Cyclic microbleeding within endometriotic lesions and subsequent inflammation may explain this finding^41^, ^42^, ^43^.

Our multivariable analysis demonstrated that there was a trend towards moderate‐to‐severe dyspareunia being associated with anxiety/depression, with the result almost reaching statistical significance (*P* = 0.08). Several other studies have reported similar observations^44^, ^45^, ^46^, ^47^. Additionally, the positive relationship between dyspareunia and history of migraine established in our study has previously been reported in two small‐scale studies^48^, ^49^. This could be explained by the previously demonstrated link between chronic conditions and dyspareunia^44^, ^50^, ^51^. This relationship may stem from the psychological effects of chronic disease, causing depression and anxiety. Alternatively, it could be related to central sensitization, triggered by the presence of chronic pain, as research has previously demonstrated a link between dyspareunia and central sensitization^52^, ^53^, ^54^, ^55^.

A 10‐year increase in age was associated with an approximately 30% reduction in the odds of moderate‐to‐severe dyspareunia in our study. Mitchell *et al*.^44^ observed a similar trend. A possible explanation for this is that treatable causes of dyspareunia are more likely to have been addressed with increasing age.

Non‐endometriotic ovarian cysts appeared to be a protective factor for moderate‐to‐severe dyspareunia. This could be explained by 61/87 (70.1%) of the cysts being functional cysts, which typically resolve within a few weeks. The group of women without non‐endometriotic ovarian cysts only included women with endometriotic ovarian cysts, which are persistent and therefore more likely to cause pain.

The main strength of this study is that it also included women with asymptomatic or mildly symptomatic endometriosis who were able to have expectant or medical management, eliminating selection bias. Furthermore, this was a large prospective study involving a consecutive sample of patients and a single examiner. Confounding factors that could also contribute to pain were adjusted for in the multivariable analyses.

Limitations of the study include the absence of histological confirmation of endometriosis; however, TVS and laparoscopy are now considered to be comparable with histology for the diagnosis of deep and ovarian endometriosis^3^. Although TVS has also demonstrated high specificity for the detection of superficial endometriosis, it is not as sensitive and our study did not focus on this subtype^56^. Other weaknesses are the lack of differentiation between superficial and deep dyspareunia and lack of exploration of other aspects of sexual or pelvic floor function. Similarly, we recognize that adenomyosis often co‐exists with endometriosis, causing overlapping symptoms^6^, ^8^. We adjusted for this in our multivariable analysis; however, examination of the association between adenomyosis and pelvic pain symptoms was beyond the scope of this study.

In conclusion, our study found that moderate‐to‐severe dysmenorrhea, menstrual dyschezia, difficulty emptying the bladder and lower EQ‐5D score were (significantly or borderline significantly) more common in women with sonographic evidence of endometriosis compared to those without, but the presence of many other pelvic pain and bowel symptoms did not vary significantly between the two groups. This will be important in reassuring women with endometriosis that a large proportion of cases can be asymptomatic. Before considering surgery, other causes of pain should be excluded and patients should be informed that surgery may not alleviate pain if the pain is not directly related to endometriosis. Further research is required to understand the impact of the number, location and size of endometriotic lesions on symptoms.

## Supporting information


**Tables S1–S15** Univariable analysis of demographic and clinical factors associated with moderate‐to‐severe premenstrual pain (Table S1), menstrual pain (dysmenorrhea) (Table S2), non‐cyclical pain (Table S3), menstrual dyschezia (Table S4), non‐menstrual dyschezia (Table S5), lower back pain (Table S6), bladder pain (Table S7), difficulty emptying bladder (Table S8), frequency of bowel movement (Table S9), urgency of bowel movement (Table S10), incomplete bowel emptying (Table S11), constipation (Table S12), menstrual rectal bleeding (Table S13), EQ Visual Analog Scale (EQ‐VAS) score (Table S14) and EuroQol‐5D‐3L (EQ‐5D) score (Table S15)
**Tables S16–S29** Multivariable analysis of factors associated with moderate‐to‐severe premenstrual pain (Table S16), menstrual pain (dysmenorrhea) (Table S17), non‐cyclical pain (Table S18), menstrual dyschezia (Table S19), non‐menstrual dyschezia (Table S20), lower back pain (Table S21), bladder pain (Table S22), difficulty emptying bladder (Table S23), urgency of bowel movement (Table S24), incomplete bowel emptying (Table S25), constipation (Table S26), menstrual rectal bleeding (Table S27), EQ Visual Analog Scale (EQ‐VAS) score (Table S28) and EuroQol‐5D‐3L (EQ‐5D) score (Table S29)

## Data Availability

The data that support the findings of this study are available from the corresponding author upon reasonable request.
