# Supplementary material for: Impact of deep or ovarian endometriosis on pelvic pain and quality of life: prospective cross‐sectional ultrasound study
Source: Ultrasound Obstet Gynecol. 2025 Jan 14;65(3):372–83. doi: 10.1002/uog.29150 (PMC11872344; doi:10.1002/uog.29150)
Supplement: Supplementary file 1 — Tables S1–S15 Univariable analysis of demographic and clinical factors associated with moderate‐to‐severe premenstrual pain (Table S1), menstrual pain (dysmenorrhea) (Table S2), non‐cyclical pain (Table S3), menstrual dyschezia (Table S4), non‐menstrual dyschezia (Table S5), lower back pain (Table S6), bladder pain (Table S7), difficulty emptying bladder (Table S8), frequency of bowel movement (Table S9), urgency of bowel movement (Table S10), incomplete bowel emptying (Table S11), constipation (Table S12), menstrual rectal bleeding (Table S13), EQ Visual Analog Scale (EQ‐VAS) score (Table S14) and EuroQol‐5D‐3L (EQ‐5D) score (Table S15) Tables S16–S29 Multivariable analysis of factors associated with moderate‐to‐severe premenstrual pain (Table S16), menstrual pain (dysmenorrhea) (Table S17), non‐cyclical pain (Table S18), menstrual dyschezia (Table S19), non‐menstrual dyschezia (Table S20), lower back pain (Table S21), bladder pain (Table S22), difficulty emptying bladder (Table S23), urgency of bowel movement (Table S24), incomplete bowel emptying (Table S25), constipation (Table S26), menstrual rectal bleeding (Table S27), EQ Visual Analog Scale (EQ‐VAS) score (Table S28) and EuroQol‐5D‐3L (EQ‐5D) score (Table S29) [file UOG-65-372-s001.docx]

**Table S1** Univariable analysis of demographic and clinical factors associated with moderate-to-severe premenstrual pain

| Variable | Category | Premenstrual pain  n/N (%) | Odds Ratio  (95% CI) | P-value |
| --- | --- | --- | --- | --- |
|  |  |  |  |  |
| Endometriosis | No | 219/342 (64.0) | 1 | **0.02** |
|  | Yes | 107/142 (75.4) | 1.72 (1.10–2.67) |  |
|  |  |  |  |  |
| Age ^(*)^ | - | - | 0.86 (0.67–1.09) | 0.21 |
|  |  |  |  |  |
| Ethnicity | White | 167/258 (64.7) | 1 | 0.42 |
|  | Black | 43/59 (72.9) | 1.46 (0.78–2.74) |  |
|  | Asian | 40/54 (74.1) | 1.56 (0.80–3.01) |  |
|  | Mixed / Other | 76/113 (67.3) | 1.12 (0.70–1.79) |  |
|  |  |  |  |  |
| BMI (kg/m^2^) ^(*)^ | - | - | 1.62 (1.11–2.35) | **0.01** |
|  |  |  |  |  |
| BMI category ^(#)^ | Normal | 140/223 (62.8) | 1 | **0.04** |
|  | Underweight | 14/18 (77.8) | 2.08 (0.66–6.51) |  |
|  | Pre-obesity | 63/96 (65.6) | 1.13 (0.69–1.87) |  |
|  | Obesity class 1 | 32/46 (69.6) | 1.36 (0.68–2.69) |  |
|  | Obesity class 2/3 | 28/32 (87.5) | 4.15 (1.41–12.2) |  |
|  |  |  |  |  |
| Smoking status | Non-smoker | 198/296 (66.9) | 1 | 0.21 |
|  | Ex-smoker | 75/118 (63.6) | 0.86 (0.55–1.40) |  |
|  | Current smoker | 53/70 (75.7) | 1.54 (0.85–2.80) |  |
|  |  |  |  |  |
| Regular periods | No | 97/127 (76.4) | 1 | **0.01** |
|  | Yes | 229/357 (64.1) | 0.55 (0.35–0.88) |  |
|  |  |  |  |  |
| Period frequency | Normal | 202/313 (64.5) | 1 | 0.14 |
|  | Infrequent | 14/23 (60.8) | 0.85 (0.36–2.04) |  |
|  | Frequent | 10/15 (66.7) | 1.10 (0.37–5.09) |  |
|  | Variable | 100/133 (75.2) | 1.67 (1.05–2.62) |  |
|  |  |  |  |  |
| Period length | Normal | 264/399 (66.2) | 1 | 0.47 |
|  | Prolonged | 16/22 (72.7) | 1.36 (0.52–3.56) |  |
|  | Variable | 46/63 (73.0) | 1.38 (0.76–2.51) |  |
|  |  |  |  |  |
| Gravidity | 0 | 156/235 (66.4) | 1 | 0.54 |
|  | 1 | 68/105 (64.8) | 0.93 (0.57–1.51) |  |
|  | 2+ | 102/144 (70.8) | 1.23 (0.78–1.93) |  |
| Parity | 0 | 210/318 (66.0) | 1 | 0.64 |
|  | 1 | 45/66 (68.2) | 1.10 (0.62–1.94) |  |
|  | 2+ | 71/100 (71.0) | 1.26 (0.77–2.06) |  |
|  |  |  |  |  |
| Prior Cesarean | 0 | 78/112 (69.6) | 1 | 0.88 |
| delivery ^(+)^ | 1 | 21/31 (67.7) | 0.92 (0.39–2.15) |  |
|  | 2+ | 17/23 (73.9) | 1.24 (0.45–3.40) |  |
|  |  |  |  |  |
| Prior vaginal | 0 | 29/42 (69.0.0) | 1 | 0.93 |
| delivery ^(+)^ | 1 | 36/50 (72) | 1.15 (0.47–2.83) |  |
|  | 2+ | 51/74 (68.9) | 0.99 (0.44–2.25) |  |
|  |  |  |  |  |
| Analgesia use | No | 40/78 (51.3) | 1 | **0.001** |
|  | Yes | 286/406 (70.4) | 2.26 (1.38–3.71) |  |
|  |  |  |  |  |
| Hormonal | No | 258/388 (66.5) | 1 | 0.42 |
| contraception | Yes | 68/96 (70.8) | 1.22 (0.75–1.99) |  |
|  |  |  |  |  |
| IBS | No | 308/463 (66.5) | 1 | 0.08 |
|  | Yes | 18/21 (85.7) | 3.02 (0.88–10.4) |  |
|  |  |  |  |  |
| IBD | No | 324/480 (67.5) | 1 | 0.47 |
|  | Yes | 2/4 (50.0) | 0.48 (0.07–3.45) |  |
|  |  |  |  |  |
| Anxiety / depression | No | 305/456 (66.9) | 1 | 0.38 |
|  | Yes | 21/28 (75.0) | 1.49 (0.62–3.57) |  |
|  |  |  |  |  |
| Fibromyalgia | No | 318/475 (66.9) | 1 | 0.20 |
|  | Yes | 8/9 (88.8) | 3.95 (0.49–31.9) |  |
|  |  |  |  |  |
| Migraine | No | 307/459 (66.9) | 1 | 0.35 |
|  | Yes | 19/25 (76.0) | 1.57 (0.61–4.01) |  |
|  |  |  |  |  |
| Adenomyosis | No | 222/342 (64.9) | 1 | 0.08 |
|  | Yes | 104/142 (73.2) | 1.48 (0.96–2.28) |  |
|  |  |  |  |  |
| Uterine fibroid(s) | No | 223/319 (69.9) | 1 | 0.10 |
|  | Yes | 103/165 (62.4) | 0.72 (0.48–1.06) |  |
|  |  |  |  |  |
| Non-endometriotic | No | 280/402 (69.7) | 1 | **0.02** |
| ovarian cyst(s) | Yes | 46/82 (56.1) | 0.56 (0.34–0.90) |  |
|  |  |  |  |  |
| Pelvic adhesions | No | 219/346 (63.3) | 1 | **0.003** |
|  | Yes | 107/138 (77.5) | 2.00 (1.27–3.16) |  |
|  |  |  |  |  |
| Dilated pelvic | No | 218/332 (65.6) | 1 | 0.07 |
| vein(s) ^(##)^ | Yes | 38/48 (79.2) | 1.99 (0.96–4.13) |  |
|  |  |  |  |  |
| Dilated fallopian | No | 317/468 (67.7) | 1 | 0.34 |
| tube(s) | Yes | 9/16 (56.3) | 0.61 (0.22–1.68) |  |
|  |  |  |  |  |

(*) Odds ratio given for 10-unit increase in predictor variable. N=484 instead of 514 as 27 excluded due to amenorrhea, and 3 excluded due to missing data. (#) N= 415 due to missing data. (+) Analysis excluding women with parity 0 (N= 166). (##) N= 380 due to missing data. CI, confidence interval. BMI, body mass index. IBS, irritable bowel syndrome. IBD, inflammatory bowel disease.

**Table S2** Univariable analysis of demographic and clinical factors associated with moderate-to-severe menstrual pain (dysmenorrhea)

| Variable | Category | Dysmenorrhea  n/N (%) | Odds Ratio  (95% CI) | P-value |
| --- | --- | --- | --- | --- |
|  |  |  |  |  |
| Endometriosis | No | 274/343 (79.9) | 1 | 0.05 |
|  | Yes | 124/142 (87.3) | 1.73 (0.99–3.04) |  |
|  |  |  |  |  |
| Age ^(*)^ | - | - | 0.60 (0.44–0.82) | **0.001** |
|  |  |  |  |  |
| Ethnicity | White | 212/258 (82.2) | 1 | 0.92 |
|  | Black | 50/59 (84.7) | 1.21 (0.55–2.62) |  |
|  | Asian | 44/55 (80.0) | 0.87 (0.42–1.81) |  |
|  | Mixed / Other | 92/113 (81.4) | 0.95 (0.54–1.68) |  |
|  |  |  |  |  |
| BMI (kg/m^2^) ^(*)^ | - | - | 1.91 (1.17–3.12) | **0.01** |
|  |  |  |  |  |
| BMI category ^(#)^ | Normal | 173/223 (77.6) | 1 | **0.04** |
|  | Underweight | 15/18 (83.3) | 1.45 (0.40–5.19) |  |
|  | Pre-obesity | 78/96 (81.2) | 1.25 (0.69–2.29) |  |
|  | Obesity class 1 | 40/47 (85.1) | 1.65 (0.70–3.91) |  |
|  | Obesity class 2/3 | 31/32 (96.9) | 8.96 (1.19–67.3) |  |
|  |  |  |  |  |
| Smoking status | Non-smoker | 237/297 (79.8) | 1 | **0.006** |
|  | Ex-smoker | 95/118 (80.5) | 1.05 (0.61–1.79) |  |
|  | Current smoker | 66/70 (94.3) | 4.18 (1.46–11.9) |  |
|  |  |  |  |  |
| Regular periods | No | 106/127 (83.5) | 1 | 0.63 |
|  | Yes | 292/358 (81.6) | 0.88 (0.51–1.50) |  |
|  |  |  |  |  |
| Period frequency | Normal | 257/314 (81.8) | 1 | 0.77 |
|  | Infrequent | 17/23 (73.9) | 0.63 (0.24–1.66) |  |
|  | Frequent | 13/15 (86.7) | 1.44 (0.32–6.56) |  |
|  | Variable | 111/133 (83.5) | 1.12 (0.65–1.92) |  |
|  |  |  |  |  |
| Period length | Normal | 329/400 (82.3) | 1 | 0.51 |
|  | Prolonged | 16/22 (72.7) | 0.58 (0.22–1.52) |  |
|  | Variable | 53/63 (84.1) | 1.14 (0.56–2.36) |  |
|  |  |  |  |  |
| Gravidity | 0 | 199/235 (84.7) | 1 | 0.31 |
|  | 1 | 85/105 (81.0) | 0.77 (0.42–1.40) |  |
|  | 2+ | 114/145 (78.6) | 0.67 (0.39–1.13) |  |
|  |  |  |  |  |
| Parity | 0 | 269/318 (84.6) | 1 | 0.13 |
|  | 1 | 53/66 (80.3) | 0.57 (0.30–1.08) |  |
|  | 2+ | 79/101 (78.2) | 0.65 (0.37–1.15) |  |
|  |  |  |  |  |
| Prior Cesarean | 0 | 90/113 (79.6) | 1 | 0.19 |
| delivery ^(+)^ | 1 | 20/31 (64.5) | 0.46 (0.20–1.10) |  |
|  | 2+ | 19/23 (82.6) | 1.21 (0.38–3.92) |  |
|  |  |  |  |  |
| Prior vaginal | 0 | 29/42 (69.0) | 1 | 0.23 |
| delivery ^(+)^ | 1 | 42/50 (84.0) | 2.35 (0.87–6.41) |  |
|  | 2+ | 58/75 (77.3) | 1.53 (0.65–3.57) |  |
|  |  |  |  |  |
| Analgesia use | No | 48/78 (61.5) | 1 | **<0.001** |
|  | Yes | 350/407 (86.0) | 3.84 (2.25–6.55) |  |
|  |  |  |  |  |
| Hormonal | No | 320/389 (82.3) | 1 | 0.82 |
| contraception | Yes | 78/96 (81.3) | 0.93 (0.53–1.66) |  |
|  |  |  |  |  |
| IBS | No | 378/464 (81.5) | 1 | 0.14 |
|  | Yes | 20/21 (95.2) | 4.55 (0.60–34.4) |  |
|  |  |  |  |  |
| IBD | No | 394/481 (81.9) | (**) | 1.00 |
|  | Yes | 4/4 (100) |  |  |
|  |  |  |  |  |
| Anxiety / depression | No | 375/457 (82.1) | 1 | 0.99 |
|  | Yes | 23/28 (82.1) | 1.01 (0.37–2.72) |  |
|  |  |  |  |  |
| Fibromyalgia | No | 389/476 (81.7) | (**) | 0.37 |
|  | Yes | 9/9 (100) |  |  |
|  |  |  |  |  |
| Migraine | No | 374/460 (81.3) | 1 | 0.10 |
|  | Yes | 24/25 (96.0) | 5.52 (0.74–41.4) |  |
|  |  |  |  |  |
| Adenomyosis | No | 278/342 (81.3) | 1 | 0.49 |
|  | Yes | 120/143 (83.9) | 1.20 (0.71–2.03) |  |
|  |  |  |  |  |
| Uterine fibroid(s) | No | 272/320 (85.0) | 1 | **0.02** |
|  | Yes | 126/165 (76.4) | 0.57 (0.36–0.91) |  |
|  |  |  |  |  |
| Non-endometriotic | No | 334/402 (83.1) | 1 | 0.20 |
| ovarian cyst(s) | Yes | 64/83 (77.1) | 0.69 (0.39–1.22) |  |
|  |  |  |  |  |
| Pelvic adhesions | No | 280/346 (80.9) | 1 | 0.30 |
|  | Yes | 118/139 (84.9) | 1.32 (0.77–2.26) |  |
|  |  |  |  |  |
| Dilated pelvic | No | 271/332 (81.6) | 1 | 0.18 |
| vein(s) ^(##)^ | Yes | 43/48 (89.6) | 1.94 (0.74–5.09) |  |
|  |  |  |  |  |
| Dilated fallopian | No | 386/469 (82.3) | 1 | 0.46 |
| tube(s) | Yes | 12/16 (75.0) | 0.65 (0.20–2.05) |  |
|  |  |  |  |  |

(*) Odds ratio given for 10-unit increase in predictor variable. N=485 instead of 514 as 27 excluded due to amenorrhea, and 2 excluded due to missing data. (#) N= 416 due to missing data. (+) Analysis excluding women with parity 0 (N= 167). (##) N= 380 due to missing data. (**) Unable to calculate odds ratio as all subjects in one category had moderate/severe pain. Analysis using Fisher’s exact test. CI, confidence interval. BMI, body mass index. IBS, irritable bowel syndrome. IBD, inflammatory bowel disease.

**Table S3** Univariable analysis of demographic and clinical factors associated with moderate-to-severe non-cyclical pain

| Variable | Category | Non-cyclical pain  n/N (%) | Odds Ratio  (95% CI) | P-value |
| --- | --- | --- | --- | --- |
|  |  |  |  |  |
| Endometriosis | No | 156/368 (42.4) | 1 | 0.39 |
|  | Yes | 68/146 (46.6) | 1.18 (0.81–1.74) |  |
|  |  |  |  |  |
| Age ^(*)^ | - | - | 0.96 (0.77–1.20) | 0.73 |
|  |  |  |  |  |
| Ethnicity | White | 120/275 (43.6) | 1 | 0.96 |
|  | Black | 26/61 (42.6) | 0.96 (0.55–1.68) |  |
|  | Asian | 27/58 (46.6) | 1.13 (0.64–1.99) |  |
|  | Mixed / Other | 51/120 (42.5) | 0.95 (0.62–1.47) |  |
|  |  |  |  |  |
| BMI (kg/m^2^) ^(*)^ | - | - | 1.19 (0.89–1.61) | 0.24 |
|  |  |  |  |  |
| BMI category ^(#)^ | Normal | 88/234 (37.6) | 1 | 0.28 |
|  | Underweight | 10/18 (55.6) | 2.07 (0.79–5.45) |  |
|  | Pre-obesity | 47/100 (47.0) | 1.47 (0.92–2.36) |  |
|  | Obesity class 1 | 24/50 (48.0) | 1.53 (0.83–2.83) |  |
|  | Obesity class 2/3 | 16/36 (44.4) | 1.33 (0.65–2.70) |  |
|  |  |  |  |  |
| Smoking status ^(##)^ | Non-smoker | 130/318 (40.9) | 1 | 0.15 |
|  | Ex-smoker | 52/120 (43.3) | 1.11 (0.72–1.69) |  |
|  | Current smoker | 39/73 (53.4) | 1.66 (0.99–2.76) |  |
|  |  |  |  |  |
| Regular periods ^(+)^ | No | 64/129 (49.6) | 1 | 0.09 |
|  | Yes | 147/358 (41.1) | 0.71 (0.47–1.06) |  |
|  |  |  |  |  |
| Period frequency | Normal | 128/314 (40.8) | 1 | 0.62 |
|  | Infrequent | 12/24 (50.0) | 1.45 (0.63–3.34) |  |
|  | Frequent | 7/15 (46.7) | 1.27 (0.45–3.59) |  |
|  | Variable | 64/134 (47.8) | 1.33 (0.88–2.00) |  |
|  | No periods | 13/27 (48.1) | 1.35 (0.61–2.97) |  |
|  |  |  |  |  |
| Period length ^(+)^ | Normal | 175/402 (43.5) | 1 | 0.79 |
|  | Prolonged | 8/22 (36.4) | 0.74 (0.30–1.81) |  |
|  | Variable | 28/63 (44.4) | 1.04 (0.61–1.77) |  |
|  |  |  |  |  |
| Gravidity | 0 | 112/255 (43.9) | 1 | 0.18 |
|  | 1 | 39/107 (36.4) | 0.73 (0.46–1.17) |  |
|  | 2+ | 73/152 (48.0) | 1.18 (0.79–1.77) |  |
| Parity | 0 | 144/339 (42.5) | 1 | 0.70 |
|  | 1 | 30/69 (43.5) | 1.04 (0.62–1.76) |  |
|  | 2+ | 50/106 (47.2) | 1.21 (0.78–1.87) |  |
|  |  |  |  |  |
| Prior Cesarean | 0 | 49/115 (42.6) | 1 | 0.20 |
| delivery ^(++)^ | 1 | 16/36 (44.4) | 1.08 (0.51–2.29) |  |
|  | 2+ | 15/24 (62.5) | 2.24 (0.91–5.55) |  |
|  |  |  |  |  |
| Prior vaginal | 0 | 26/45 (57.8) | 1 | 0.09 |
| delivery ^(++)^ | 1 | 19/53 (35.8) | 0.41 (0.18–0.92) |  |
|  | 2+ | 35/77 (45.5) | 0.61 (0.29–1.28) |  |
|  |  |  |  |  |
| Analgesia use | No | 22/82 (26.8) | 1 | **0.001** |
|  | Yes | 202/432 (46.8) | 2.40 (1.42–4.04) |  |
|  |  |  |  |  |
| Hormonal | No | 164/394 (41.6) | 1 | 0.11 |
| contraception | Yes | 60/120 (50.0) | 1.40 (0.93–2.11) |  |
|  |  |  |  |  |
| IBS | No | 209/492 (42.5) | 1 | **0.02** |
|  | Yes | 15/22 (68.2) | 2.90 (1.16–7.24) |  |
|  |  |  |  |  |
| IBD | No | 222/510 (43.5) | 1 | 0.80 |
|  | Yes | 2/4 (50.0) | 1.30 (0.18–9.28) |  |
|  |  |  |  |  |
| Anxiety / depression | No | 208/481 (43.2) | 1 | 0.59 |
|  | Yes | 16/33 (48.5) | 1.24 (0.61–2.50) |  |
|  |  |  |  |  |
| Fibromyalgia | No | 217/504 (43.1) | 1 | 0.11 |
|  | Yes | 7/10 (70.0) | 3.09 (0.79–12.1) |  |
|  |  |  |  |  |
| Migraine | No | 310/488 (63.5) | 1 | 0.28 |
|  | Yes | 14/26 (53.8) | 1.54 (0.70–3.41) |  |
|  |  |  |  |  |
| Adenomyosis | No | 153/364 (42.0) | 1 | 0.27 |
|  | Yes | 71/150 (47.3) | 1.24 (0.85–1.82) |  |
|  |  |  |  |  |
| Uterine fibroid(s) | No | 156/343 (45.5) | 1 | 0.22 |
|  | Yes | 68/171 (39.8) | 0.79 (0.55–1.15) |  |
|  |  |  |  |  |
| Non-endometriotic | No | 194/427 (45.4) | 1 | 0.06 |
| ovarian cyst(s) | Yes | 30/87 (34.5) | 0.63 (0.39–1.03) |  |
|  |  |  |  |  |
| Pelvic adhesions | No | 156/371 (42.0) | 1 | 0.26 |
|  | Yes | 68/143 (47.6) | 1.25 (0.84–1.84) |  |
|  |  |  |  |  |
| Dilated pelvic | No | 153/354 (43.2) | 1 | 0.30 |
| vein(s) ^(###)^ | Yes | 25/49 (51.0) | 1.37 (0.75–2.49) |  |
|  |  |  |  |  |
| Dilated fallopian | No | 216/498 (43.4) | 1 | 0.60 |
| tube(s) | Yes | 8/16 (50.0) | 1.31 (0.48–3.53) |  |
|  |  |  |  |  |

(*) Odds ratio given for 10-unit increase in predictor variable. (#) N= 438 due to missing data. (##) N=511 due to missing data. (+) Women with no periods excluded (N= 487). (++) Analysis excluding women with parity 0 (N= 175). (###) N= 403 due to missing data. CI, confidence interval. BMI, body mass index. IBS, irritable bowel syndrome. IBD, inflammatory bowel disease.

**Table S4** Univariable analysis of demographic and clinical factors associated with moderate-to-severe menstrual dyschezia

| Variable | Category | Menstrual dyschezia  n/N (%) | Odds Ratio  (95% CI) | P-value |
| --- | --- | --- | --- | --- |
|  |  |  |  |  |
| Endometriosis | No | 97/343 (28.3) | 1 | **<0.001** |
|  | Yes | 68/142 (47.9) | 2.33 (1.56–3.49) |  |
|  |  |  |  |  |
| Age ^(*)^ | - | - | 0.66 (0.52–0.85) | **0.001** |
|  |  |  |  |  |
| Ethnicity | White | 81/258 (31.4) | 1 | 0.53 |
|  | Black | 24/59 (40.7) | 1.50 (0.84–2.68) |  |
|  | Asian | 19/55 (34.5) | 1.15 (0.62–2.13) |  |
|  | Mixed / Other | 41/113 (36.3) | 1.24 (0.78–1.98) |  |
|  |  |  |  |  |
| BMI (kg/m^2^) ^(*)^ | - | - | 1.14 (0.83–1.57) | 0.41 |
|  |  |  |  |  |
| BMI category ^(#)^ | Normal | 66/223 (29.6) | 1 | 0.49 |
|  | Underweight | 8/18 (44.4) | 1.90 (0.72–5.04) |  |
|  | Pre-obesity | 36/96 (37.5) | 1.43 (0.86–2.36) |  |
|  | Obesity class 1 | 17/46 (37.0) | 1.39 (0.72–2.71) |  |
|  | Obesity class 2/3 | 10/32 (31.3) | 1.08 (0.49–2.41) |  |
|  |  |  |  |  |
| Smoking status | Non-smoker | 99/296 (33.4) | 1 | 0.07 |
|  | Ex-smoker | 34/118 (28.8) | 0.81 (0.51–1.28) |  |
|  | Current smoker | 32/71 (45.1) | 1.63 (0.96–2.76) |  |
|  |  |  |  |  |
| Regular periods | No | 43/129 (33.3) | 1 | 0.85 |
|  | Yes | 122/356 (34.3) | 1.04 (0.68–1.60) |  |
|  |  |  |  |  |
| Period frequency | Normal | 110/312 (35.3) | 1 | 0.28 |
|  | Infrequent | 11/24 (45.8) | 1.55 (0.67–3.58) |  |
|  | Frequent | 6/15 (40.0) | 1.22 (0.42–3.53) |  |
|  | Variable | 38/134 (28.4) | 0.73 (0.47–1.13) |  |
|  |  |  |  |  |
| Period length | Normal | 144/400 (36.0) | 1 | **0.02** |
|  | Prolonged | 9/22 (40.9) | 1.23 (0.51–2.95) |  |
|  | Variable | 12/63 (19.0) | 0.42 (0.22–0.81) |  |
|  |  |  |  |  |
| Gravidity | 0 | 84/237 (35.4) | 1 | 0.78 |
|  | 1 | 35/104 (33.7) | 0.92 (0.57–1.50) |  |
|  | 2+ | 46/144 (31.9) | 0.85 (0.55–1.33) |  |
| Parity | 0 | 111/320 (34.7) | 1 | 0.83 |
|  | 1 | 20/65 (30.8) | 0.84 (0.47, 1.49) |  |
|  | 2+ | 34/100 (34.0) | 0.97 (0.60, 1.56) |  |
|  |  |  |  |  |
| Prior Cesarean | 0 | 37/112 (33.0) | 1 | 0.30 |
| delivery ^(+)^ | 1 | 7/30 (23.3) | 0.62 (0.25, 1.57) |  |
|  | 2+ | 10/23 (43.5) | 1.56 (0.63, 3.89) |  |
|  |  |  |  |  |
| Prior vaginal | 0 | 14/42 (33.3) | 1 | 0.98 |
| delivery ^(+)^ | 1 | 16/48 (33.3) | 1.00 (0.42–2.41) |  |
|  | 2+ | 24/75 (32.0) | 0.94 (0.42–2.10) |  |
|  |  |  |  |  |
| Analgesia use | No | 18/78 (23.1) | 1 | **0.03** |
|  | Yes | 147/407 (36.1) | 1.88 (1.07–3.31) |  |
|  |  |  |  |  |
| Hormonal | No | 129/388 (33.2) | 1 | 0.47 |
| contraception | Yes | 36/97 (37.1) | 1.18 (0.75–1.88) |  |
|  |  |  |  |  |
| IBS | No | 155/464 (33.4) | 1 | 0.18 |
|  | Yes | 10/21 (47.6) | 1.81 (0.75–4.36) |  |
|  |  |  |  |  |
| IBD | No | 164/481 (34.1) | 1 | 0.70 |
|  | Yes | 1/4 (25.0) | 0.64 (0.07–6.24) |  |
|  |  |  |  |  |
| Anxiety / depression | No | 156/456 (34.2) | 1 | 0.73 |
|  | Yes | 9/29 (31.0) | 0.87 (0.38–1.95) |  |
|  |  |  |  |  |
| Fibromyalgia | No | 158/476 (33.2) | 1 | **0.02** |
|  | Yes | 7/9 (77.8) | 7.04 (1.44–34.3) |  |
|  |  |  |  |  |
| Migraine | No | 150/460 (32.6) | 1 | **0.007** |
|  | Yes | 15/25 (60.0) | 3.10 (1.36–7.06) |  |
|  |  |  |  |  |
| Adenomyosis | No | 113/343 (32.9) | 1 | 0.44 |
|  | Yes | 52/142 (36.6) | 1.18 (0.78–1.77) |  |
|  |  |  |  |  |
| Uterine fibroid(s) | No | 116/319 (36.4) | 1 | 0.13 |
|  | Yes | 49/166 (29.5) | 0.73 (0.49–1.10) |  |
|  |  |  |  |  |
| Non-endometriotic | No | 147/403 (36.5) | 1 | **0.01** |
| ovarian cyst(s) | Yes | 18/82 (22.0) | 0.49 (0.28–0.86) |  |
|  |  |  |  |  |
| Pelvic adhesions | No | 105/346 (30.3) | 1 | **0.007** |
|  | Yes | 60/139 (43.2) | 1.74 (1.16–2.62) |  |
|  |  |  |  |  |
| Dilated pelvic | No | 118/331 (35.6) | 1 | 0.42 |
| vein(s) ^(##)^ | Yes | 20/48 (41.7) | 1.29 (0.70–2.39) |  |
|  |  |  |  |  |
| Dilated fallopian | No | 158/469 (33.7) | 1 | 0.41 |
| tube(s) | Yes | 7/16 (43.8) | 1.53 (0.56–4.19) |  |
|  |  |  |  |  |

(*) Odds ratio given for 10-unit increase in predictor variable. N=485 instead of 514 as 27 excluded due to amenorrhoea, and 2 excluded due to missing data. (#) N= 415 due to missing data. (+) Analysis excluding women with parity 0 (N= 165). (##) N= 379 due to missing data. CI, confidence interval. BMI, body mass index. IBS, irritable bowel syndrome. IBD, inflammatory bowel disease.

**Table S5** Univariable analysis of demographic and clinical factors associated with moderate-to-severe non-menstrual dyschezia

| Variable | Category | Non-menstrual Dyschezia  n/N (%) | Odds Ratio  (95% CI) | P-value |
| --- | --- | --- | --- | --- |
|  |  |  |  |  |
| Endometriosis | No | 74/367 (20.2) | 1 | 0.20 |
|  | Yes | 37/146 (25.3) | 1.34 (0.86–2.11) |  |
|  |  |  |  |  |
| Age ^(*)^ | - | - | 0.78 (0.60–1.03) | 0.08 |
|  |  |  |  |  |
| Ethnicity | White | 56/274 (20.4) | 1 | 0.79 |
|  | Black | 12/61 (19.7) | 0.95 (0.48–1.91) |  |
|  | Asian | 14/58 (24.1) | 1.24 (0.63–2.42) |  |
|  | Mixed / Other | 29/120 (24.2) | 1.24 (0.74–2.07) |  |
|  |  |  |  |  |
| BMI (kg/m^2^) ^(*)^ | - | - | 1.24 (0.87–1.75) | 0.23 |
|  |  |  |  |  |
| BMI category ^(#)^ | Normal | 44/233 (18.9) | 1 | 0.81 |
|  | Underweight | 5/18 (27.8) | 1.65 (0.56–4.87) |  |
|  | Pre-obesity | 18/100 (18.0) | 0.94 (0.51–1.73) |  |
|  | Obesity class 1 | 10/50 (20.0) | 1.07 (0.50–2.31) |  |
|  | Obesity class 2/3 | 9/36 (25.0) | 1.43 (0.63–3.26) |  |
|  |  |  |  |  |
| Smoking status ^(##)^ | Non-smoker | 66/317 (20.8) | 1 | 0.39 |
|  | Ex-smoker | 23/120 (19.2) | 0.90 (0.53–1.53) |  |
|  | Current smoker | 20/73 (27.4) | 1.44 (0.80–2.57) |  |
|  |  |  |  |  |
| Regular periods ^(+)^ | No | 30/129 (23.3) | 1 | 0.38 |
|  | Yes | 70/357 (19.6) | 0.80 (0.50–1.31) |  |
|  |  |  |  |  |
| Period frequency | Normal | 62/313 (19.8) | 1 | **0.04** |
|  | Infrequent | 8/24 (33.3) | 2.02 (0.83–4.94) |  |
|  | Frequent | 1/15 (6.7) | 0.29 (0.04–2.24) |  |
|  | Variable | 29/134 (21.6) | 1.12 (0.68–1.84) |  |
|  | No periods | 11/27 (40.7) | 2.78 (1.23–6.30) |  |
|  |  |  |  |  |
| Period length ^(+)^ | Normal | 85/401 (21.2) | 1 | 0.59 |
|  | Prolonged | 5/22 (22.7) | 1.09 (0.39–3.05) |  |
|  | Variable | 10/63 (15.9) | 0.70 (0.34–1.44) |  |
|  |  |  |  |  |
| Gravidity | 0 | 54/255 (21.2) | 1 | 0.74 |
|  | 1 | 21/106 (19.8) | 0.92 (0.52–1.62) |  |
|  | 2+ | 36/152 (23.7) | 1.16 (0.72–1.87) |  |
| Parity | 0 | 74/339 (21.8) | 1 | **0.04** |
|  | 1 | 8/68 (11.8) | 0.48 (0.22–1.04) |  |
|  | 2+ | 29/106 (27.4) | 1.34 (0.82–2.22) |  |
|  |  |  |  |  |
| Prior Cesarean | 0 | 23/114 (20.2) | 1 | 0.10 |
| delivery ^(++)^ | 1 | 5/36 (13.9) | 0.64 (0.22–1.82) |  |
|  | 2+ | 9/24 (37.5) | 2.37 (0.92–6.10) |  |
|  |  |  |  |  |
| Prior vaginal | 0 | 9/45 (20.0) | 1 | 0.59 |
| delivery ^(++)^ | 1 | 9/52 (17.3) | 0.84 (0.30–2.33) |  |
|  | 2+ | 19/77 (24.7) | 1.31 (0.54–3.21) |  |
|  |  |  |  |  |
| Analgesia use | No | 9/82 (11.0) | 1 | **0.01** |
|  | Yes | 102/431 (23.7) | 2.51 (1.22–5.20) |  |
|  |  |  |  |  |
| Hormonal | No | 79/393 (20.1) | 1 | 0.13 |
| contraception | Yes | 32/120 (26.7) | 1.45 (0.90–2.32) |  |
|  |  |  |  |  |
| IBS | No | 103/491 (21.0) | 1 | 0.09 |
|  | Yes | 8/22 (36.4) | 2.15 (0.88–5.27) |  |
|  |  |  |  |  |
| IBD | No | 108/509 (21.2) | 1 | **0.04** |
|  | Yes | 3/4 (75.0) | 11.3 (1.15–108) |  |
|  |  |  |  |  |
| Anxiety / depression | No | 99/480 (20.6) | 1 | **0.04** |
|  | Yes | 12/33 (36.4) | 2.20 (1.05–4.62) |  |
|  |  |  |  |  |
| Fibromyalgia | No | 106/503 (21.1) | 1 | **0.04** |
|  | Yes | 5/10 (50.0) | 3.75 (1.06–13.2) |  |
|  |  |  |  |  |
| Migraine | No | 104/487 (21.4) | 1 | 0.50 |
|  | Yes | 7/26 (26.9) | 1.36 (0.56–3.31) |  |
|  |  |  |  |  |
| Adenomyosis | No | 76/363 (20.9) | 1 | 0.55 |
|  | Yes | 35/150 (23.3) | 1.15 (0.73–1.81) |  |
|  |  |  |  |  |
| Uterine fibroid(s) | No | 82/342 (24.0) | 1 | 0.07 |
|  | Yes | 29/171 (17.0) | 0.65 (0.40–1.04) |  |
|  |  |  |  |  |
| Non-endometriotic | No | 97/427 (22.7) | 1 | 0.19 |
| ovarian cyst(s) | Yes | 14/86 (16.3) | 0.66 (0.36–1.22) |  |
|  |  |  |  |  |
| Pelvic adhesions | No | 73/370 (19.7) | 1 | 0.09 |
|  | Yes | 38/143 (26.6) | 1.47 (0.94–2.31) |  |
|  |  |  |  |  |
| Dilated pelvic | No | 75/354 (21.2) | 1 | 0.25 |
| vein(s) ^(###)^ | Yes | 14/49 (28.6) | 1.49 (0.76–2.91) |  |
|  |  |  |  |  |
| Dilated fallopian | No | 109/497 (21.9) | 1 | 0.38 |
| tube(s) | Yes | 2/16 (12.5) | 0.51 (0.11–2.27) |  |
|  |  |  |  |  |

(*) Odds ratio given for 10-unit increase in predictor variable. N= 513 rather than 514 due to missing data for one patient. (#) N= 437 due to missing data. (##) N=510 due to missing data. (+) Women with no periods excluded (N= 486). (++) Analysis excluding women with parity 0 (N= 174). (###) N= 403 due to missing data. CI, confidence interval. BMI, body mass index. IBS, irritable bowel syndrome. IBD, inflammatory bowel disease.

**Table S6** Univariable analysis of demographic and clinical factors associated with moderate-to-severe lower back pain

| Variable | Category | Pain  n/N (%) | Odds Ratio  (95% CI) | P-value |
| --- | --- | --- | --- | --- |
|  |  |  |  |  |
| Endometriosis | No | 234/366 (63.9) | 1 | 0.26 |
|  | Yes | 101/146 (69.2) | 1.27 (0.84–1.91) |  |
|  |  |  |  |  |
| Age ^(*)^ | - | - | 0.84 (0.67–1.06) | 0.15 |
|  |  |  |  |  |
| Ethnicity | White | 178/275 (64.7) | 1 | 0.56 |
|  | Black | 38/60 (63.3) | 0.94 (0.53–1.68) |  |
|  | Asian | 42/57 (73.7) | 1.53 (0.81–2.89) |  |
|  | Mixed / Other | 77/120 (64.2) | 0.98 (0.62–1.53) |  |
|  |  |  |  |  |
| BMI (kg/m^2^) ^(*)^ | - | - | 2.05 (1.40–2.98) | **<0.001** |
|  |  |  |  |  |
| BMI category ^(#)^ | Normal | 138/234 (59.0) | 1 | **0.004** |
|  | Underweight | 11/18 (61.1) | 1.09 (0.41–2.92) |  |
|  | Pre-obesity | 65/100 (65.0) | 1.29 (0.79–2.10) |  |
|  | Obesity class 1 | 38/49 (77.6) | 2.40 (1.17–4.94) |  |
|  | Obesity class 2/3 | 31/36 (86.1) | 4.31 (1.62–11.5) |  |
|  |  |  |  |  |
| Smoking status ^(##)^ | Non-smoker | 191/316 (60.4) | 1 | **0.004** |
|  | Ex-smoker | 83/120 (69.2) | 1.47 (0.94–2.30) |  |
|  | Current smoker | 58/73 (79.5) | 2.53 (1.37–4.66) |  |
|  |  |  |  |  |
| Regular periods ^(+)^ | No | 86/129 (66.7) | 1 | 0.63 |
|  | Yes | 229/356 (64.3) | 0.90 (0.59–1.38) |  |
|  |  |  |  |  |
| Period frequency | Normal | 204/313 (65.2) | 1 | 0.75 |
|  | Infrequent | 14/24 (58.3) | 0.75 (0.32–1.73) |  |
|  | Frequent | 11/15 (73.3) | 1.47 (0.46–4.72) |  |
|  | Variable | 86/133 (64.7) | 0.98 (0.64–1.50) |  |
|  | No periods | 20/27 (74.1) | 1.53 (0.63–3.72) |  |
|  |  |  |  |  |
| Period length ^(+)^ | Normal | 253/400 (63.3) | 1 | 0.19 |
|  | Prolonged | 15/22 (68.2) | 1.25 (0.50–3.12) |  |
|  | Variable | 47/63 (74.6) | 1.71 (0.93–3.11) |  |
|  |  |  |  |  |
| Gravidity | 0 | 169/255 (66.3) | 1 | 0.61 |
|  | 1 | 65/106 (61.3) | 0.81 (0.50–1.29) |  |
|  | 2+ | 101/151 (66.9) | 1.03 (0.67–1.58) |  |
| Parity | 0 | 221/339 (65.2) | 1 | 0.65 |
|  | 1 | 42/68 (61.8) | 0.86 (0.50–1.48) |  |
|  | 2+ | 72/105 (68.6) | 1.16 (0.73–1.86) |  |
|  |  |  |  |  |
| Prior Cesarean | 0 | 73/113 (64.6) | 1 | 0.10 |
| delivery ^(++)^ | 1 | 21/36 (58.3) | 0.77 (0.36–1.65) |  |
|  | 2+ | 20/24 (83.3) | 2.74 (0.88–8.57) |  |
|  |  |  |  |  |
| Prior vaginal | 0 | 32/45 (71.1) | 1 | 0.61 |
| delivery ^(++)^ | 1 | 32/52 (61.5) | 0.65 (0.28–1.53) |  |
|  | 2+ | 50/76 (65.8) | 0.78 (0.35–1.74) |  |
|  |  |  |  |  |
| Analgesia use | No | 33/82 (40.2) | 1 | **<0.001** |
|  | Yes | 302/430 (70.2) | 3.50 (2.15–5.70) |  |
|  |  |  |  |  |
| Hormonal | No | 250/392 (63.8) | 1 | 0.16 |
| contraception | Yes | 85/120 (70.8) | 1.38 (0.88–2.15) |  |
|  |  |  |  |  |
| IBS | No | 317/490 (64.7) | 1 | 0.11 |
|  | Yes | 18/22 (81.8) | 2.46 (0.82–7.37) |  |
|  |  |  |  |  |
| IBD | No | 332/508 (65.4) | 1 | 0.69 |
|  | Yes | 3/4 (75.0) | 1.59 (0.16–15.4) |  |
|  |  |  |  |  |
| Anxiety / depression | No | 313/480 (65.2) | 1 | 0.68 |
|  | Yes | 22/32 (68.8) | 1.17 (0.54–2.54) |  |
|  |  |  |  |  |
| Fibromyalgia | No | 327/502 (65.1) | 1 | 0.34 |
|  | Yes | 8/10 (80.0) | 2.14 (0.45–10.2) |  |
|  |  |  |  |  |
| Migraine | No | 314/486 (64.6) | 1 | 0.10 |
|  | Yes | 21/26 (80.8) | 2.30 (0.85–6.21) |  |
|  |  |  |  |  |
| Adenomyosis | No | 228/364 (62.6) | 1 | **0.04** |
|  | Yes | 107/148 (72.3) | 1.56 (1.02–2.36 |  |
|  |  |  |  |  |
| Uterine fibroid(s) | No | 239/342 (69.9) | 1 | **0.003** |
|  | Yes | 96/170 (56.5) | 0.56 (0.38–0.82) |  |
|  |  |  |  |  |
| Non-endometriotic | No | 281/426 (66.0) | 1 | 0.57 |
| ovarian cyst(s) | Yes | 54/86 (62.8) | 0.87 (0.54–1.41) |  |
|  |  |  |  |  |
| Pelvic adhesions | No | 232/370 (62.7) | 1 | **0.04** |
|  | Yes | 103/142 (72.5) | 1.57 (1.03–2.40) |  |
|  |  |  |  |  |
| Dilated pelvic | No | 232/354 (65.5) | 1 | 0.80 |
| vein(s) ^(###)^ | Yes | 33/49 (67.3) | 1.08 (0.57–2.05) |  |
|  |  |  |  |  |
| Dilated fallopian | No | 325/496 (65.5) | 1 | 0.80 |
| tube(s) | Yes | 10/16 (62.5) | 0.88 (0.31–2.45) |  |
|  |  |  |  |  |
|  |  |  |  |  |

(*) Odds ratio given for 10-unit increase in predictor variable. N= 512 rather than 514 due to missing data for two patients. (#) N= 437 due to missing data. (##) N=509 due to missing data. (+) Women with no periods excluded (N= 485). (++) Analysis excluding women with parity 0 (N= 173). (###) N= 403 due to missing data. CI, confidence interval. BMI, body mass index. IBS, irritable bowel syndrome. IBD, inflammatory bowel disease.

**Table S7** Univariable analysis of demographic and clinical factors associated with moderate-to-severe bladder pain

| Variable | Category | Bladder pain  n/N (%) | Odds Ratio  (95% CI) | P-value |
| --- | --- | --- | --- | --- |
|  |  |  |  |  |
| Endometriosis | No | 60/368 (16.3) | 1 | **0.01** |
|  | Yes | 38/146 (26.0) | 1.81 (1.14–2.87) |  |
|  |  |  |  |  |
| Age ^(*)^ | - | - | 0.82 (0.62–1.09) | 0.17 |
|  |  |  |  |  |
| Ethnicity | White | 43/275 (15.6) | 1 | 0.20 |
|  | Black | 13/61 (21.3) | 1.46 (0.73–2.92) |  |
|  | Asian | 14/58 (24.1) | 1.72 (0.87–3.40) |  |
|  | Mixed / Other | 28/120 (23.3) | 1.64 (0.96–2.80) |  |
|  |  |  |  |  |
| BMI (kg/m^2^) ^(*)^ | - | - | 1.33 (0.93–1.89) | 0.12 |
|  |  |  |  |  |
| BMI category ^(#)^ | Normal | 35/234 (15.0) | 1 | 0.47 |
|  | Underweight | 5/18 (27.8) | 2.19 (0.73–6.2) |  |
|  | Pre-obesity | 21/100 (21.0) | 1.51 (0.83–2.76) |  |
|  | Obesity class 1 | 9/50 (18.0) | 1.25 (0.56–2.79) |  |
|  | Obesity class 2/3 | 8/36 (22.2) | 1.62 (0.68–3.85) |  |
|  |  |  |  |  |
| Smoking status ^(##)^ | Non-smoker | 63/318 (19.8) | 1 | 0.63 |
|  | Ex-smoker | 19/120 (15.8) | 0.76 (0.43–1.34) |  |
|  | Current smoker | 14/73 (19.2) | 0.96 (0.50–1.83) |  |
|  |  |  |  |  |
| Regular periods ^(+)^ | No | 21/129 (16.3) | 1 | 0.41 |
|  | Yes | 70/358 (19.6) | 1.25 (0.73–2.14) |  |
|  |  |  |  |  |
| Period frequency | Normal | 61/314 (19.4) | 1 | 0.68 |
|  | Infrequent | 6/24 (25.0) | 1.38 (0.53–3.62) |  |
|  | Frequent | 2/15 (13.3) | 0.64 (0.14–2.90) |  |
|  | Variable | 22/134 (16.4) | 0.81 (0.48–1.39) |  |
|  | No periods | 7/27 (25.9) | 1.45 (0.59–3.59) |  |
|  |  |  |  |  |
| Period length ^(+)^ | Normal | 76/402 (18.9) | 1 | 0.75 |
|  | Prolonged | 5/22 (22.7) | 1.26 (0.45–3.53) |  |
|  | Variable | 10/63 (15.9) | 0.81 (0.39–1.66) |  |
|  |  |  |  |  |
| Gravidity | 0 | 55/255 (21.6) | 1 | 0.30 |
|  | 1  2+ | 16/107 (15.0)  27/152 (17.8) | 0.64 (0.35–1.18)  0.79 (0.47–1.31) |  |
| Parity | 0 | 69/339 (20.4) | 1 | 0.20 |
|  | 1 | 8/69 (11.6) | 0.51 (0.23–1.12) |  |
|  | 2+ | 21/106 (19.8) | 0.97 (0.56–1.67) |  |
|  |  |  |  |  |
| Prior Cesarean | 0 | 19/115 (16.5) | 1 | 0.78 |
| delivery ^(++)^ | 1 | 5/36 (13.9) | 0.81 (0.28–2.36) |  |
|  | 2+ | 5/24 (20.8) | 1.33 (0.44–4.00) |  |
|  |  |  |  |  |
| Prior vaginal | 0 | 8/45 (17.8) | 1 | 0.44 |
| delivery ^(++)^ | 1 | 6/53 (11.3) | 0.59 (0.19–1.85) |  |
|  | 2+ | 15/77 (19.5) | 1.12 (0.43–2.89) |  |
|  |  |  |  |  |
| Analgesia use | No | 8/82 (9.8) | 1 | **0.02** |
|  | Yes | 90/432 (20.8) | 2.43 (1.13–5.23) |  |
|  |  |  |  |  |
| Hormonal | No | 64/394 (16.2) | 1 | **0.004** |
| contraception | Yes | 34/120 (28.3) | 2.04 (1.26–3.29) |  |
|  |  |  |  |  |
| IBS | No | 92/492 (18.7) | 1 | 0.32 |
|  | Yes | 6/22 (27.3) | 1.63 (0.92–4.28) |  |
|  |  |  |  |  |
| IBD | No | 98/510 (19.2) | (**) | 1.00 |
|  | Yes | 0/4 (0.0) |  |  |
|  |  |  |  |  |
| Anxiety / depression | No | 91/481 (18.9) | 1 | 0.75 |
|  | Yes | 7/33 (21.2) | 1.15 (0.49–2.74) |  |
|  |  |  |  |  |
| Fibromyalgia | No | 91/504 (18.1) | 1 | **0.001** |
|  | Yes | 7/10 (70.0) | 10.6 (2.69–41.7) |  |
|  |  |  |  |  |
| Migraine | No | 93/488 (19.1) | 1 | 0.98 |
|  | Yes | 5/26 (19.2) | 1.01 (0.37–2.75) |  |
|  |  |  |  |  |
| Adenomyosis | No | 63/364 (17.3) | 1 | 0.12 |
|  | Yes | 35/150 (23.3) | 1.45 (0.91–2.32) |  |
|  |  |  |  |  |
| Uterine fibroid(s) | No | 69/343 (20.1) | 1 | 0.39 |
|  | Yes | 29/171 (17.0) | 0.81 (0.50–1.31) |  |
|  |  |  |  |  |
| Non-endometriotic | No | 89/427 (20.8) | 1 | **0.03** |
| ovarian cyst(s) | Yes | 9/87 (10.3) | 0.44 (0.21–0.91) |  |
|  |  |  |  |  |
| Pelvic adhesions | No | 60/371 (16.2) | 1 | **0.008** |
|  | Yes | 38/143 (26.6) | 1.88 (1.18–2.98) |  |
|  |  |  |  |  |
| Dilated pelvic | No | 67/354 (18.9) | 1 | 0.12 |
| vein(s) ^(###)^ | Yes | 14/49 (28.6) | 1.71 (0.87–3.36) |  |
|  |  |  |  |  |
| Dilated fallopian | No | 96/498 (19.3) | 1 | 0.50 |
| tube(s) | Yes | 2/16 (12.5) | 0.60 (0.13–2.68) |  |
|  |  |  |  |  |

(*) Odds ratio given for 10-unit increase in predictor variable. (#) N= 438 due to missing data. (##) N=511 due to missing data. (+) Women with no periods excluded (N= 487). (++) Analysis excluding women with parity 0 (N= 175). (###) N= 403 due to missing data. (**) Unable to calculate odds ratio as no subjects in one category had moderate/severe pain. Analysis using Fisher’s exact test. CI, confidence interval. BMI, body mass index. IBS, irritable bowel syndrome. IBD, inflammatory bowel disease.

**Table S8** Univariable analysis of demographic and clinical factors associated with moderate-to-severe difficulties emptying bladder

| Variable | Category | Difficulty emptying bladder  n/N (%) | Odds Ratio  (95% CI) | P-value |
| --- | --- | --- | --- | --- |
|  |  |  |  |  |
| Endometriosis | No | 39/368 (10.6) | 1 | **<0.001** |
|  | Yes | 34/146 (23.3) | 2.56 (1.54–4.25) |  |
|  |  |  |  |  |
| Age ^(*)^ | - | - | 1.06 (0.78–1.46) | 0.70 |
|  |  |  |  |  |
| Ethnicity | White | 33/275 (12.0) | 1 | 0.10 |
|  | Black | 6/61 (9.8) | 0.80 (0.32–2.00) |  |
|  | Asian | 9/58 (15.5) | 1.35 (0.61–2.99) |  |
|  | Mixed / Other | 25/120 (20.8) | 1.93 (1.09–3.42) |  |
|  |  |  |  |  |
| BMI (kg/m^2^) ^(*)^ | - | - | 1.30 (0.88–1.91) | 0.19 |
|  |  |  |  |  |
| BMI category ^(#)^ | Normal | 24/234 (10.3) | 1 | 0.11 |
|  | Underweight | 5/18 (27.8) | 3.37 (1.10–10.3) |  |
|  | Pre-obesity | 19/100 (19.0) | 2.05 (1.07–3.95) |  |
|  | Obesity class 1 | 8/50 (16.0) | 1.67 (0.70–3.96) |  |
|  | Obesity class 2/3 | 4/36 (11.1) | 1.09 (0.36–3.36) |  |
|  |  |  |  |  |
| Smoking status ^(##)^ | Non-smoker | 45/318 (14.2) | 1 | 0.98 |
|  | Ex-smoker | 17/120 (14.2) | 1.00 (0.55–1.83) |  |
|  | Current smoker | 11/73 (15.1) | 1.08 (0.53–2.20) |  |
|  |  |  |  |  |
| Regular periods ^(+)^ | No | 11/129 (8.5) | 1 | **0.04** |
|  | Yes | 57/358 (15.9) | 2.03 (1.03–4.01) |  |
|  |  |  |  |  |
| Period frequency | Normal | 48/314 (15.3) | 1 | 0.20 |
|  | Infrequent | 6/24 (25.0) | 1.85 (0.70–4.89) |  |
|  | Frequent | 2/15 (13.3) | 0.85 (0.19–3.90) |  |
|  | Variable | 12/134 (9.0) | 0.55 (0.28–1.06) |  |
|  | No periods | 5/27 (18.5) | 1.26 (0.45–3.49) |  |
|  |  |  |  |  |
| Period length ^(+)^ | Normal | 52/402 (12.9) | 1 | 0.09 |
|  | Prolonged | 7/22 (31.8) | 3.14 (1.22–8.07) |  |
|  | Variable | 9/63 (14.3) | 1.12 (0.52–0.52) |  |
|  |  |  |  |  |
| Gravidity | 0 | 34/255 (13.3) | 1 | 0.16 |
|  | 1 | 11/107 (10.3) | 0.74 (0.36–1.53) |  |
|  | 2+ | 28/152 (18.4) | 1.47 (0.85–2.53) |  |
| Parity | 0 | 45/339 (13.3) | 1 | 0.06 |
|  | 1 | 6/69 (8.7) | 0.62 (0.25–1.52) |  |
|  | 2+ | 22/106 (20.8) | 1.71 (0.97–3.01) |  |
|  |  |  |  |  |
| Prior Cesarean | 0 | 22/115 (19.1) | 1 | 0.24 |
| delivery ^(++)^ | 1 | 3/36 (8.3) | 0.38 (0.11–1.37) |  |
|  | 2+ | 3/24 (12.5) | 1.60 (0.17–2.21) |  |
|  |  |  |  |  |
| Prior vaginal | 0 | 4/45 (8.9) | 1 | 0.06 |
| delivery ^(++)^ | 1 | 6/53 (11.3) | 1.31 (0.35–4.97) |  |
|  | 2+ | 18/77 (23.4) | 3.13 (0.99–9.92) |  |
|  |  |  |  |  |
| Analgesia use | No | 7/82 (8.5) | 1 | 0.11 |
|  | Yes | 66/432 (15.2) | 1.93 (0.85–4.38) |  |
|  |  |  |  |  |
| Hormonal | No | 56/394 (14.2) | 1 | 0.99 |
| contraception | Yes | 17/120 (14.1) | 1.00 (0.55–1.79) |  |
|  |  |  |  |  |
| IBS | No | 68/492 (13.8) | 1 | 0.25 |
|  | Yes | 5/22 (22.7) | 1.83 (0.66–5.13) |  |
|  |  |  |  |  |
| IBD | No | 73/510 (14.3) | (**) | 1.00 |
|  | Yes | 0/4 (0.0) |  |  |
|  |  |  |  |  |
| Anxiety / depression | No | 70/481 (14.6) | 1 | 0.39 |
|  | Yes | 3/33 (9.1) | 0.59 (0.17–1.98) |  |
|  |  |  |  |  |
| Fibromyalgia | No | 68/504 (13.5) | 1 | **0.004** |
|  | Yes | 5/10 (50.0) | 6.41 (1.81–22.7) |  |
|  |  |  |  |  |
| Migraine | No | 67/488 (13.7) | 1 | 0.19 |
|  | Yes | 6/26 (23.1) | 1.89 (0.73–4.86) |  |
|  |  |  |  |  |
| Adenomyosis | No | 46/364 (12.6) | 1 | 0.12 |
|  | Yes | 27/150 (18.0) | 1.52 (0.90–2.55) |  |
|  |  |  |  |  |
| Uterine fibroid(s) | No | 47/343 (13.7) | 1 | 0.65 |
|  | Yes | 26/171 (15.2) | 1.13 (0.67–1.90) |  |
|  |  |  |  |  |
| Non-endometriotic | No | 61/427 (14.3) | 1 | 0.90 |
| ovarian cyst(s) | Yes | 12/87 (13.8) | 0.96 (0.49–1.87) |  |
|  |  |  |  |  |
| Pelvic adhesions | No | 42/371 (11.3) | 1 | **0.003** |
|  | Yes | 31/143 (21.7) | 2.17 (1.30–3.62) |  |
|  |  |  |  |  |
| Dilated pelvic | No | 51/354 (14.4) | 1 | 0.47 |
| vein(s) ^(###)^ | Yes | 9/49 (18.4) | 1.34 (0.61–2.92) |  |
|  |  |  |  |  |
| Dilated fallopian | No | 71/498 (14.3) | 1 | 0.84 |
| tube(s) | Yes | 2/16 (12.5) | 0.86 (0.19–3.86) |  |
|  |  |  |  |  |

(*) Odds ratio given for 10-unit increase in predictor variable. (#) N= 438 due to missing data. (##) N=511 due to missing data. (+) Women with no periods excluded (N= 487). (++) Analysis excluding women with parity 0 (N= 175). (###) N= 403 due to missing data. (**) Unable to calculate odds ratio as no subjects in one category had moderate/severe difficulties. Analysis using Fisher’s exact test. CI, confidence interval. BMI, body mass index. IBS, irritable bowel syndrome. IBD, inflammatory bowel disease.

**Table S9** Univariable analysis of demographic and clinical factors associated with moderate-to-severe frequency of bowel movements

| Variable | Category | Frequent bowel movements  n/N (%) | Odds Ratio  (95% CI) | P-value |
| --- | --- | --- | --- | --- |
|  |  |  |  |  |
| Endometriosis | No | 277/368 (75.3) | 1 | 0.99 |
|  | Yes | 110/146 (75.3) | 1.00 (0.64–1.57) |  |
|  |  |  |  |  |
| Age ^(*)^ | - | - | 0.78 (0.60–1.01) | 0.06 |
|  |  |  |  |  |
| Ethnicity | White | 211/275 (76.7) | 1 | 0.63 |
|  | Black | 46 /61 (75.4) | 0.93 (0.49–1.78) |  |
|  | Asian | 45/58 (77.6) | 1.05 (0.53–2.07) |  |
|  | Mixed / Other | 85/120 (70.8) | 0.74 (0.45–1.19) |  |
|  |  |  |  |  |
| BMI (kg/m^2^) ^(*)^ | - | - | 1.33 (0.91–1.94) | 0.14 |
|  |  |  |  |  |
| BMI category ^(#)^ | Normal | 179/234 (76.5) | 1 | 0.38 |
|  | Underweight | 13/18 (72.2) | 0.80 (0.27–2.34) |  |
|  | Pre-obesity | 71/100 (71.0) | 0.75 (0.44–1.27) |  |
|  | Obesity class 1 | 40/50 (80.0) | 1.23 (0.58–2.62) |  |
|  | Obesity class 2/3 | 31/36 (86.1) | 1.91 (0.71–5.14) |  |
|  |  |  |  |  |
| Smoking status ^(##)^ | Non-smoker | 234/318 (73.6) | 1 | 0.37 |
|  | Ex-smoker | 96/120 (80.0) | 1.44 (0.86–2.40) |  |
|  | Current smoker | 55/73 (75.3) | 1.10 (0.60–1.97) |  |
|  |  |  |  |  |
| Regular periods ^(+)^ | No | 94/129 (72.9) | 1 | 0.48 |
|  | Yes | 272/358 (76.0) | 1.18 (0.75–1.86) |  |
|  |  |  |  |  |
| Period frequency | Normal | 241/314 (76.8) | 1 | 0.81 |
|  | Infrequent | 18/24 (75.0) | 0.91 (0.35–2.37) |  |
|  | Frequent | 10/15 (66.7) | 0.61 (0.20–1.83) |  |
|  | Variable | 97/134 (72.4) | 0.79 (0.50–1.26) |  |
|  | No periods | 21/27 (77.8) | 1.06 (0.41–2.73) |  |
|  |  |  |  |  |
| Period length ^(+)^ | Normal | 300/402 (74.6) | 1 | 0.72 |
|  | Prolonged | 18/22 (81.8) | 1.53 (0.51–4.63) |  |
|  | Variable | 48/63 (76.2) | 1.09 (0.58–2.03) |  |
|  |  |  |  |  |
| Gravidity | 0 | 200/255 (78.4) | 1 | 0.15 |
|  | 1 | 81/107 (75.7) | 0.86 (0.50–1.46) |  |
|  | 2+ | 106/152 (69.7) | 0.63 (0.40–1.00) |  |
| Parity | 0 | 262/339 (77.3) | 1 | 0.05 |
|  | 1 | 55/69 (79.7) | 1.15 (0.61–2.19) |  |
|  | 2+ | 70/106 (66.0) | 0.57 (0.36–0.92) |  |
|  |  |  |  |  |
| Prior Cesarean | 0 | 77/115 (67.0) | 1 | 0.16 |
| delivery ^(++)^ | 1 | 28/36 (77.8) | 1.73 (0.72–4.15) |  |
|  | 2+ | 20/24 (83.3) | 2.47 (0.79–7.73) |  |
|  |  |  |  |  |
| Prior vaginal | 0 | 38/45 (84.4) | 1 | **0.003** |
| delivery ^(++)^ | 1 | 42/53 (79.2) | 0.70 (0.25–2.00) |  |
|  | 2+ | 45/77 (58.4) | 0.26 (0.10–0.65) |  |
|  |  |  |  |  |
| Analgesia use | No | 60/82 (73.2) | 1 | 0.65 |
|  | Yes | 327/432 (75.7) | 1.14 (0.67–1.95) |  |
|  |  |  |  |  |
| Hormonal | No | 294/394 (74.6) | 1 | 0.52 |
| contraception | Yes | 93/120 (77.5) | 1.17 (0.72–1.90) |  |
|  |  |  |  |  |
| IBS | No | 369/492 (75.0) | 1 | 0.47 |
|  | Yes | 18/22 (81.8) | 1.50 (0.50–4.52) |  |
|  |  |  |  |  |
| IBD | No | 383/510 (75.1) | (**) | 0.58 |
|  | Yes | 4/4 (100) |  |  |
|  |  |  |  |  |
| Anxiety / depression | No | 361/481 (75.0) | 1 | 0.63 |
|  | Yes | 26/33 (78.8) | 1.23 (0.52–2.92) |  |
|  |  |  |  |  |
| Fibromyalgia | No | 379/504 (75.2) | 1 | 0.73 |
|  | Yes | 8/10 (80.0) | 1.32 (0.28–6.29) |  |
|  |  |  |  |  |
| Migraine | No | 368/488 (75.4) | 1 | 0.79 |
|  | Yes | 19/26 (73.1) | 0.89 (0.36–2.16) |  |
|  |  |  |  |  |
| Adenomyosis | No | 277/364 (76.1) | 1 | 0.51 |
|  | Yes | 110/150 (73.3) | 0.86 (0.56–1.33) |  |
|  |  |  |  |  |
| Uterine fibroid(s) | No | 263/343 (76.7) | 1 | 0.30 |
|  | Yes | 124/171 (72.5) | 0.80 (0.53–1.22) |  |
|  |  |  |  |  |
| Non-endometriotic | No | 320/427 (74.9) | 1 | 0.68 |
| ovarian cyst(s) | Yes | 67/87 (77.0) | 1.12 (0.65–1.93) |  |
|  |  |  |  |  |
| Pelvic adhesions | No | 284/371 (76.5) | 1 | 0.29 |
|  | Yes | 103/143 (72.0) | 0.79 (0.51–1.22) |  |
|  |  |  |  |  |
| Dilated pelvic | No | 260/354 (73.4) | 1 | 0.13 |
| vein(s) ^(###)^ | Yes | 41/49 (83.7) | 1.85 (0.84–4.10) |  |
|  |  |  |  |  |
| Dilated fallopian | No | 376/498 (75.5) | 1 | 0.54 |
| tube(s) | Yes | 11/16 (68.8) | 0.71 (0.24–2.10) |  |
|  |  |  |  |  |

Following multivariable analysis, only vaginal deliveries remained associated with the presence of moderate to severe frequency of bowel movements (*P*=0.003). (*) Odds ratio given for 10-unit increase in predictor variable. (#) N= 438 due to missing data. (##) N=511 due to missing data. (+) Women with no periods excluded (N= 487). (++) Analysis excluding women with parity 0 (N= 175). (###) N= 403 due to missing data. (**) Unable to calculate odds ratio as all subjects in one category had moderate/severe frequency of movements. Analysis using Fisher’s exact test. CI, confidence interval. BMI, body mass index. IBS, irritable bowel syndrome. IBD, inflammatory bowel disease.

**Table S10** Univariable analysis of demographic and clinical factors associated with moderate-to-severe urgency of bowel movements

| Variable | Category | Urgent movement  n/N (%) | Odds Ratio  (95% CI) | P-value |
| --- | --- | --- | --- | --- |
|  |  |  |  |  |
| Endometriosis | No | 120/368 (32.6) | 1 | 0.28 |
|  | Yes | 55/146 (37.7) | 1.25 (0.84–1.86) |  |
|  |  |  |  |  |
| Age ^(*)^ | - | - | 0.99 (0.79–1.26) | 0.96 |
|  |  |  |  |  |
| Ethnicity | White | 105/275 (38.2) | 1 | 0.07 |
|  | Black | 19/61 (31.1) | 0.73 (0.40–1.33) |  |
|  | Asian | 21/58 (36.2) | 0.92 (0.51–1.65) |  |
|  | Mixed / Other | 30/120 (25.0) | 0.54 (0.33–0.87) |  |
|  |  |  |  |  |
| BMI (kg/m^2^) ^(*)^ | - | - | 1.56 (1.14–2.13) | **0.005** |
|  |  |  |  |  |
| BMI category ^(#)^ | Normal | 62/234 (26.5) | 1 | **0.04** |
|  | Underweight | 9/18 (50.0) | 2.77 (1.05–7.31) |  |
|  | Pre-obesity | 36/100 (36.0) | 1.56 (0.95–2.57) |  |
|  | Obesity class 1 | 20/50 (40.0) | 1.85 (0.98–3.49) |  |
|  | Obesity class 2/3 | 16/36 (44.4) | 2.22 (1.08–4.55) |  |
|  |  |  |  |  |
| Smoking status ^(##)^ | Non-smoker | 98/318 (30.8) | 1 | 0.12 |
|  | Ex-smoker | 49/120 (40.8) | 1.55 (1.00–2.39) |  |
|  | Current smoker | 27/73 (37.0) | 1.32 (0.77–2.24) |  |
|  |  |  |  |  |
| Regular periods ^(+)^ | No | 41/129 (31.8) | 1 | 0.72 |
|  | Yes | 120/358 (33.5) | 1.08 (0.70–1.66) |  |
|  |  |  |  |  |
| Period frequency | Normal | 104/314 (33.1) | 1 | **0.02** |
|  | Infrequent | 12/24 (50.0) | 2.02 (0.88–4.65) |  |
|  | Frequent | 8/15 (53.3) | 2.31 (0.81–6.53) |  |
|  | Variable | 37/134 (27.6) | 0.77 (0.49–1.20) |  |
|  | No periods | 14/27 (51.9) | 2.17 (0.99–4.79) |  |
|  |  |  |  |  |
| Period length ^(+)^ | Normal | 134/402 (33.3) | 1 | 0.96 |
|  | Prolonged | 7/22 (31.8) | 0.93 (0.37–3.34) |  |
|  | Variable | 20/63 (31.7) | 0.93 (0.53–1.64) |  |
|  |  |  |  |  |
| Gravidity | 0 | 85/255 (33.3) | 1 | 0.15 |
|  | 1 | 30/107 (28.0) | 0.78 (0.47–1.28) |  |
|  | 2+ | 60/152 (39.5) | 1.30 (0.86–1.98) |  |
| Parity | 0 | 110/339 (32.4) | 1 | 0.11 |
|  | 1 | 20/69 (2.09) | 0.85 (0.48–1.50) |  |
|  | 2+ | 45/106 (42.5) | 1.54 (0.98–2.40) |  |
|  |  |  |  |  |
| Prior Cesarean | 0 | 44/115 (38.3) | 1 | 0.33 |
| delivery ^(++)^ | 1 | 10/36 (27.8) | 0.62 (0.27–1.41) |  |
|  | 2+ | 11/24 (45.8) | 1.37 (0.56–3.31) |  |
|  |  |  |  |  |
| Prior vaginal | 0 | 15/45 (33.3) | 1 | 0.73 |
| delivery ^(++)^ | 1 | 19/53 (35.8) | 1.12 (0.48–2.58) |  |
|  | 2+ | 31/77 (40.3) | 1.35 (0.62–2.91) |  |
|  |  |  |  |  |
| Analgesia use | No | 18/82 (22.0) | 1 | **0.01** |
|  | Yes | 157/432 (36.3) | 2.03 (1.16–3.55) |  |
|  |  |  |  |  |
| Hormonal | No | 133/394 (33.8) | 1 | 0.80 |
| contraception | Yes | 42/120 (35.0) | 1.06 (0.69–1.62) |  |
|  |  |  |  |  |
| IBS | No | 163/492 (33.1) | 1 | **0.04** |
|  | Yes | 12/22 (54.5) | 2.42 (1.02–5.72) |  |
|  |  |  |  |  |
| IBD | No | 172/510 (33.7) | 1 | 0.13 |
|  | Yes | 3/4 (75.0) | 5.90 (0.61–57.1) |  |
|  |  |  |  |  |
| Anxiety / depression | No | 160/481 (33.3) | 1 | 0.16 |
|  | Yes | 15/33 (45.5) | 1.67 (0.82–3.40) |  |
|  |  |  |  |  |
| Fibromyalgia | No | 167/504 (33.1) | 1 | **0.009** |
|  | Yes | 8/10 (80.0) | 8.07 (1.70–38.4) |  |
|  |  |  |  |  |
| Migraine | No | 167/488 (34.2) | 1 | 0.72 |
|  | Yes | 8/26 (30.8) | 0.85 (0.36–2.01) |  |
|  |  |  |  |  |
| Adenomyosis | No | 124/364 (34.1) | 1 | 0.99 |
|  | Yes | 51/150 (34.0) | 1.00 (0.67–1.49) |  |
|  |  |  |  |  |
| Uterine fibroid(s) | No | 118/343 (34.4) | 1 | 0.81 |
|  | Yes | 57/171 (33.3) | 0.95 (0.65–1.41) |  |
|  |  |  |  |  |
| Non-endometriotic | No | 149/427 (34.9) | 1 | 0.37 |
| ovarian cyst(s) | Yes | 26/87 (29.9) | 0.80 (0.48–1.31) |  |
|  |  |  |  |  |
| Pelvic adhesions | No | 122/371 (32.9) | 1 | 0.37 |
|  | Yes | 53/143 (37.1) | 1.20 (0.80–0.61) |  |
|  |  |  |  |  |
| Dilated pelvic | No | 127/354 (35.9) | 1 | 0.20 |
| vein(s) ^(###)^ | Yes | 13/49 (26.5) | 0.65 (0.33–1.26) |  |
|  |  |  |  |  |
| Dilated fallopian | No | 171/498 (34.3) | 1 | 0.44 |
| tube(s) | Yes | 4/16 (25.0) | 0.64 (0.20–2.01) |  |
|  |  |  |  |  |

(*) Odds ratio given for 10-unit increase in predictor variable. (#) N= 438 due to missing data. (##) N=511 due to missing data. (+) Women with no periods excluded (N= 487). (++) Analysis excluding women with parity 0 (N= 175). (###) N= 403 due to missing data. CI, confidence interval. BMI, body mass index. IBS, irritable bowel syndrome. IBD, inflammatory bowel disease.

**Table S11** Univariable analysis of demographic and clinical factors associated with moderate-to-severe incomplete bowel emptying

| Variable | Category | Incomplete bowel emptying  n/N (%) | Odds Ratio  (95% CI) | P-value |
| --- | --- | --- | --- | --- |
|  |  |  |  |  |
| Endometriosis | No | 111/368 (30.2) | 1 | **0.04** |
|  | Yes | 58/146 (39.7) | 1.53 (1.02–2.27) |  |
|  |  |  |  |  |
| Age ^(*)^ | - | - | 1.00 (0.79–1.26) | 0.98 |
|  |  |  |  |  |
| Ethnicity | White | 95/275 (34.5) | 1 | 0.75 |
|  | Black | 21/61 (34.4) | 0.99 (0.55–1.78) |  |
|  | Asian | 17/58 (29.3) | 0.79 (0.42–1.46) |  |
|  | Mixed / Other | 36/120 (30.0) | 0.81 (0.51–1.29) |  |
|  |  |  |  |  |
| BMI (kg/m^2^) ^(*)^ | - | - | 1.66 (1.21–2.28) | **0.002** |
|  |  |  |  |  |
| BMI category ^(#)^ | Normal | 69/234 (29.5) | 1 | 0.15 |
|  | Underweight | 5/18 (27.8) | 0.92 (0.32–2.68) |  |
|  | Pre-obesity | 30/100 (30.0) | 1.02 (0.61–1.71) |  |
|  | Obesity class 1 | 19/50 (38.0) | 1.17 (0.78–2.77) |  |
|  | Obesity class 2/3 | 18/36 (50.0) | 2.39 (1.17–4.86) |  |
|  |  |  |  |  |
| Smoking status ^(##)^ | Non-smoker | 94/318 (29.6) | 1 | 0.12 |
|  | Ex-smoker | 47/120 (39.2) | 1.53 (0.99–2.38) |  |
|  | Current smoker | 27/73 (37.0) | 1.40 (0.82–2.38) |  |
|  |  |  |  |  |
| Regular periods ^(+)^ | No | 47/129 (36.4) | 1 | 0.19 |
|  | Yes | 108/358 (30.2) | 0.75 (0.49–1.15) |  |
|  |  |  |  |  |
| Period frequency | Normal | 92/314 (29.3) | 1 | 0.12 |
|  | Infrequent | 10/24 (41.7) | 1.72 (0.74–4.02) |  |
|  | Frequent | 5/15 (33.3) | 1.21 (0.40–3.63) |  |
|  | Variable | 48/134 (35.8) | 1.34 (0.88–2.07) |  |
|  | No periods | 14/27 (51.9) | 2.60 (1.18–5.74) |  |
|  |  |  |  |  |
| Period length ^(+)^ | Normal | 130/402 (32.3) | 1 | 0.83 |
|  | Prolonged | 7/22 (31.8) | 0.98 (0.39–2.45) |  |
|  | Variable | 18/63 (28.6) | 0.84 (0.47–1.50) |  |
|  |  |  |  |  |
| Gravidity | 0 | 84/255 (32.9) | 1 | 0.39 |
|  | 1 | 30/107 (28.0) | 0.79 (0.48–1.30) |  |
|  | 2+ | 55/152 (36.2) | 0.79 (0.76–1.76) |  |
| Parity | 0 | 113/339 (33.3) | 1 | 0.22 |
|  | 1 | 17/69 (24.6) | 0.65 (0.36–1.18) |  |
|  | 2+ | 39/106 (36.8) | 1.16 (0.74–1.83) |  |
|  |  |  |  |  |
| Prior Cesarean | 0 | 39/115 (33.9) | 1 | 0.59 |
| delivery ^(++)^ | 1 | 9/36 (25.0) | 0.65 (0.28–1.52) |  |
|  | 2+ | 8/24 (33.3) | 0.97 (0.38–2.47) |  |
|  |  |  |  |  |
| Prior vaginal | 0 | 12/45 (26.7) | 1 | 0.36 |
| delivery ^(++)^ | 1 | 15/53 (28.3) | 1.09 (0.45–2.65) |  |
|  | 2+ | 29/77 (37.7) | 1.66 (0.74–3.72) |  |
|  |  |  |  |  |
| Analgesia use | No | 22/82 (26.8) | 1 | 0.21 |
|  | Yes | 147/432 (34.0) | 1.41 (0.83–2.38) |  |
|  |  |  |  |  |
| Hormonal | No | 125/394 (31.7) | 1 | 0.31 |
| contraception | Yes | 44/120 (36.7) | 1.25 (0.81–1.91) |  |
|  |  |  |  |  |
| IBS | No | 158/492 (32.1) | 1 | 0.09 |
|  | Yes | 11/22 (50.0) | 2.11 (0.90–4.98) |  |
|  |  |  |  |  |
| IBD | No | 167/510 (32.7) | 1 | 0.47 |
|  | Yes | 2/4 (50.0) | 2.05 (0.29–14.7) |  |
|  |  |  |  |  |
| Anxiety / depression | No | 153/481 (31.8) | 1 | 0.05 |
|  | Yes | 16/33 (48.5) | 2.02 (0.99–4.10) |  |
|  |  |  |  |  |
| Fibromyalgia | No | 163/504 (32.3) | 1 | 0.08 |
|  | Yes | 6/10 (60.0) | 3.14 (0.87–11.3) |  |
|  |  |  |  |  |
| Migraine | No | 158/488 (32.4) | 1 | 0.30 |
|  | Yes | 11/26 (42.3) | 1.53 (0.69–3.41) |  |
|  |  |  |  |  |
| Adenomyosis | No | 115/364 (31.6) | 1 | 0.33 |
|  | Yes | 54/150 (36.0) | 1.22 (0.82–1.82) |  |
|  |  |  |  |  |
| Uterine fibroid(s) | No | 109/343 (31.8) | 1 | 0.45 |
|  | Yes | 60/171 (35.1) | 1.16 (0.79–1.71) |  |
|  |  |  |  |  |
| Non-endometriotic | No | 143/427 (33.5) | 1 | 0.52 |
| ovarian cyst(s) | Yes | 26/87 (29.9) | 0.85 (0.51–1.40) |  |
|  |  |  |  |  |
| Pelvic adhesions | No | 113/371 (30.5) | 1 | 0.06 |
|  | Yes | 56/143 (39.2) | 1.47 (0.98–2.20) |  |
|  |  |  |  |  |
| Dilated pelvic | No | 118/354 (33.3) | 1 | 0.85 |
| vein(s) ^(###)^ | Yes | 17/49 (34.7) | 1.06 (0.57–1.99) |  |
|  |  |  |  |  |
| Dilated fallopian | No | 163/498 (32.7) | 1 | 0.69 |
| tube(s) | Yes | 6/16 (37.5) | 1.23 (0.44–3.45) |  |
|  |  |  |  |  |
|  |  |  |  |  |

(*) Odds ratio given for 10-unit increase in predictor variable. (#) N= 438 due to missing data. (##) N=511 due to missing data. (+) Women with no periods excluded (N= 487). (++) Analysis excluding women with parity 0 (N= 175). (###) N= 403 due to missing data. CI, confidence interval. BMI, body mass index. IBS, irritable bowel syndrome. IBD, inflammatory bowel disease.

**Table S12** Univariable analysis of demographic and clinical factors associated with moderate-to-severe constipation

| Variable | Category | Constipation  n/N (%) | Odds Ratio  (95% CI) | P-value |
| --- | --- | --- | --- | --- |
|  |  |  |  |  |
| Endometriosis | No | 130/368 (35.3) | 1 | 0.10 |
|  | Yes | 63/146 (43.2) | 1.39 (0.94–2.05) |  |
|  |  |  |  |  |
| Age ^(*)^ | - | - | 0.89 (0.70–1.11) | 0.30 |
|  |  |  |  |  |
| Ethnicity | White | 107/275 (38.9) | 1 | 0.14 |
|  | Black | 27/61 (44.3) | 1.25 (0.71–2.18) |  |
|  | Asian | 24/58 (41.4) | 1.11 (0.62–1.97) |  |
|  | Mixed / Other | 35/120 (29.2) | 0.65 (0.41–1.03) |  |
|  |  |  |  |  |
| BMI (kg/m^2^) ^(*)^ | - | - | 1.17 (0.87–1.59) | 0.30 |
|  |  |  |  |  |
| BMI category ^(#)^ | Normal | 77/234 (32.9) | 1 | 0.44 |
|  | Underweight | 8/18 (44.4) | 1.63 (0.62–4.30) |  |
|  | Pre-obesity | 37/100 (37.0) | 1.20 (0.73–1.95) |  |
|  | Obesity class 1 | 23/50 (46.0) | 1.74 (0.93–3.23) |  |
|  | Obesity class 2/3 | 14/36 (38.9) | 1.30 (0.63–2.67) |  |
|  |  |  |  |  |
| Smoking status ^(##)^ | Non-smoker | 113/318 (35.5) | 1 | 0.39 |
|  | Ex-smoker | 47/120 (39.2) | 1.17 (0.76–1.80) |  |
|  | Current smoker | 32/73 (43.8) | 1.42 (0.84–2.37) |  |
|  |  |  |  |  |
| Regular periods ^(+)^ | No | 54/129 (41.9) | 1 | 0.22 |
|  | Yes | 128/358 (35.8) | 0.77 (0.51–1.17) |  |
|  |  |  |  |  |
| Period frequency | Normal | 114/314 (36.3) | 1 | 0.50 |
|  | Infrequent | 10/24 (41.7) | 1.25 (0.54–2.91) |  |
|  | Frequent | 3/15 (20.0) | 0.44 (0.12–1.59) |  |
|  | Variable | 55/134 (41.0) | 1.22 (0.81–1.85) |  |
|  | No periods | 11/27 (40.7) | 1.21 (0.54–2.68) |  |
|  |  |  |  |  |
| Period length ^(+)^ | Normal | 155/402 (38.6) | 1 | 0.13 |
|  | Prolonged | 9/22 (40.9) | 1.10 (0.46–2.64) |  |
|  | Variable | 18/63 (28.6) | 0.64 (0.36–1.14) |  |
|  |  |  |  |  |
| Gravidity | 0 | 95/255 (37.3) | 1 | 0.98 |
|  | 1 | 40/107 (37.4) | 1.01 (0.63–1.60) |  |
|  | 2+ | 58/152 (38.2) | 1.04 (0.69–1.57) |  |
| Parity | 0 | 126/339 (37.2) | 1 | 0.36 |
|  | 1 | 22/69 (31.9) | 0.79 (0.46–1.37) |  |
|  | 2+ | 45/106 (42.5) | 1.25 (0.80–1.94) |  |
|  |  |  |  |  |
| Prior Cesarean | 0 | 43/115 (37.4) | 1 | 0.41 |
| delivery ^(++)^ | 1 | 12/36 (33.3) | 0.84 (0.38–1.84) |  |
|  | 2+ | 12/24 (50.0) | 1.67 (0.69–4.06) |  |
|  |  |  |  |  |
| Prior vaginal | 0 | 17/45 (37.8) | 1 | 0.88 |
| delivery ^(++)^ | 1 | 19/53 (35.8) | 0.92 (0.40–2.10) |  |
|  | 2+ | 31/77 (40.3) | 1.11 (0.52–2.36) |  |
|  |  |  |  |  |
| Analgesia use | No | 22/82 (26.8) | 1 | **0.03** |
|  | Yes | 171/432 (39.6) | 1.79 (1.06–3.02) |  |
|  |  |  |  |  |
| Hormonal | No | 151/394 (38.3) | 1 | 0.51 |
| contraception | Yes | 42/120 (35.0) | 0.87 (0.57–1.33) |  |
|  |  |  |  |  |
| IBS | No | 183/492 (37.2) | 1 | 0.44 |
|  | Yes | 10/22 (45.5) | 1.41 (0.60–3.32) |  |
|  |  |  |  |  |
| IBD | No | 191/510 (37.5) | 1 | 0.61 |
|  | Yes | 2/4 (50.0) | 1.67 (0.23–12.0) |  |
|  |  |  |  |  |
| Anxiety / depression | No | 170/481 (35.3) | 1 | **<0.001** |
|  | Yes | 23/33 (69.7) | 4.21 (1.96–9.05) |  |
|  |  |  |  |  |
| Fibromyalgia | No | 187/504 (37.1) | 1 | 0.15 |
|  | Yes | 6/10 (60.0) | 2.54 (0.71–9.13) |  |
|  |  |  |  |  |
| Migraine | No | 175/488 (35.9) | 1 | **0.001** |
|  | Yes | 18/26 (69.2) | 4.02 (1.71–9.44) |  |
|  |  |  |  |  |
| Adenomyosis | No | 125/364 (34.3) | 1 | **0.02** |
|  | Yes | 68/150 (45.3) | 1.59 (1.08–2.33) |  |
|  |  |  |  |  |
| Uterine fibroid(s) | No | 125/343 (36.4) | 1 | 0.46 |
|  | Yes | 68/171 (39.8) | 1.15 (0.79–1.68) |  |
|  |  |  |  |  |
| Non-endometriotic | No | 169/427 (39.6) | 1 | **0.04** |
| ovarian cyst(s) | Yes | 24/87 (27.6) | 0.58 (0.35–0.97) |  |
|  |  |  |  |  |
| Pelvic adhesions | No | 127/371 (34.2) | 1 | **0.01** |
|  | Yes | 66/143 (46.2) | 1.65 (1.11–2.44) |  |
|  |  |  |  |  |
| Dilated pelvic | No | 136/354 (38.4) | 1 | 0.62 |
| vein(s) ^(###)^ | Yes | 17/49 (34.7) | 0.85 (0.46–1.59) |  |
|  |  |  |  |  |
| Dilated fallopian | No | 187/498 (37.6) | 1 | 0.99 |
| tube(s) | Yes | 6/16 (37.5) | 1.00 (0.36–2.79) |  |
|  |  |  |  |  |

(*) Odds ratio given for 10-unit increase in predictor variable. (#) N= 438 due to missing data. (##) N=511 due to missing data. (+) Women with no periods excluded (N= 487). (++) Analysis excluding women with parity 0 (N= 175). (###) N= 403 due to missing data. CI, confidence interval. BMI, body mass index. IBS, irritable bowel syndrome. IBD, inflammatory bowel disease.

**Table S13** Univariable analysis of demographic and clinical factors associated with moderate-to-severe menstrual hematochezia

| Variable | Category | Menstrual hematochezia  n/N (%) | Odds Ratio  (95% CI) | P-value |
| --- | --- | --- | --- | --- |
|  |  |  |  |  |
| Endometriosis | No | 34/345 (9.9) | 1 | 0.08 |
|  | Yes | 22/142 (15.5) | 1.68 (0.94–2.98) |  |
|  |  |  |  |  |
| Age ^(*)^ | - | - | 0.83 (0.58–1.19) | 0.31 |
|  |  |  |  |  |
| Ethnicity | White | 32/259 (12.4) | 1 | 0.65 |
|  | Black | 6/59 (10.2) | 0.80 (0.32–2.02) |  |
|  | Asian | 8/55 (14.5) | 1.21 (0.52–2.79) |  |
|  | Mixed / Other | 10/114 (8.8) | 0.68 (0.32–1.44) |  |
|  |  |  |  |  |
| BMI (kg/m^2^) ^(*)^ | - | - | 1.24 (0.80–1.93) | 0.33 |
|  |  |  |  |  |
| BMI ^(#)^ | Normal | 24/224 (10.7) | 1 | 0.95 |
|  | Underweight | 2/18 (11.1) | 1.04 (0.23–4.81) |  |
|  | Pre-obesity | 11/96 (11.5) | 1.08 (0.51–2.30) |  |
|  | Obesity class 1 | 6/47 (12.8) | 1.22 (0.47–3.17) |  |
|  | Obesity class 2/3 | 5/32 (15.6) | 1.54 (0.54–4.38) |  |
|  |  |  |  |  |
| Smoking status | Non-smoker | 32/298 (10.7) | 1 | 0.33 |
|  | Ex-smoker | 12/118 (10.2) | 0.94 (0.47–1.90) |  |
|  | Current smoker | 12/71 (16.9) | 1.69 (0.82–3.47) |  |
|  |  |  |  |  |
| Regular periods | No | 15/129 (11.6) | 1 | 0.96 |
|  | Yes | 41/358 (11.5) | 0.98 (0.52–1.84) |  |
|  |  |  |  |  |
| Period frequency | Normal | 35/314 (11.1) | 1 | 0.88 |
|  | Infrequent | 4/24 (16.7) | 1.59 (0.52–4.93) |  |
|  | Frequent | 2/15 (13.3) | 1.23 (0.27–5.66) |  |
|  | Variable | 15/134 (11.2) | 1.00 (0.53–1.91) |  |
|  |  |  |  |  |
| Period length | Normal | 50/402 (12.4) | 1 | 0.28 |
|  | Prolonged | 1/22 (4.5) | 0.34 (0.04–2.55) |  |
|  | Variable | 5/63 (7.9) | 0.61 (0.23–1.59) |  |
|  |  |  |  |  |
| Gravidity | 0 | 31/237 (13.1) | 1 | 0.32 |
|  | 1 | 8/105 (7.6) | 0.55 (0.24–1.24) |  |
|  | 2+ | 17/145 (11.7) | 0.88 (0.47–1.66) |  |
| Parity | 0 | 35/320 (10.9) | 1 | 0.86 |
|  | 1 | 8/66 (12.1) | 1.12 (0.50–2.55) |  |
|  | 2+ | 13/101 (12.9) | 1.20 (0.61–2.37) |  |
|  |  |  |  |  |
| Prior Cesarean | 0 | 13/113 (11.5) | 1 | 0.11 |
| delivery ^(+)^ | 1 | 2/31 (6.5) | 0.53 (0.11–2.48) |  |
|  | 2+ | 6/23 (26.1) | 2.71 (0.91–8.11) |  |
|  |  |  |  |  |
| Prior vaginal | 0 | 8/42 (19.0) | 1 | 0.33 |
| delivery ^(+)^ | 1 | 6/50 (12.0) | 0.58 (0.18–1.83) |  |
|  | 2+ | 7/75 (9.3) | 0.44 (0.15–1.31) |  |
|  |  |  |  |  |
| Analgesia use | No | 6/78 (7.7) | 1 | 0.26 |
|  | Yes | 50/409 (12.2) | 1.67 (0.69–4.04) |  |
|  |  |  |  |  |
| Hormonal | No | 44/390 (11.3) | 1 | 0.76 |
| contraception | Yes | 12/97 (12.4) | 1.11 (0.56–2.19) |  |
|  |  |  |  |  |
| IBS | No | 53/466 (11.4) | 1 | 0.68 |
|  | Yes | 3/21 (14.3) | 1.30 (0.37–4.56) |  |
|  |  |  |  |  |
| IBD | No | 56/483 (11.6) | (**) | 1.00 |
|  | Yes | 0/4 (0.0) |  |  |
|  |  |  |  |  |
| Anxiety / depression | No | 51/458 (11.1) | 1 | 0.32 |
|  | Yes | 5/29 (17.2) | 1.66 (0.61–4.55) |  |
|  |  |  |  |  |
| Fibromyalgia | No | 53/478 (11.1) | 1 | 0.05 |
|  | Yes | 3/9 (33.3) | 4.00 (0.97–16.5) |  |
|  |  |  |  |  |
| Migraine | No | 51/462 (11.0) | 1 | 0.18 |
|  | Yes | 5/25 (20.0) | 2.01 (0.72–5.60) |  |
|  |  |  |  |  |
| Adenomyosis | No | 33/344 (9.6) | 1 | **0.04** |
|  | Yes | 23/143 (16.1) | 1.81 (1.02–3.20) |  |
|  |  |  |  |  |
| Uterine fibroid(s) | No | 40/321 (12.5) | 1 | 0.36 |
|  | Yes | 16/166 (9.6) | 0.75 (0.41–1.38) |  |
|  |  |  |  |  |
| Non-endometriotic | No | 49/404 (12.1) | 1 | 0.34 |
| ovarian cyst(s) | Yes | 7/83 (8.4) | 0.67 (0.29–1.53) |  |
|  |  |  |  |  |
| Pelvic adhesions | No | 33/348 (9.5) | 1 | **0.03** |
|  | Yes | 23/139 (16.5) | 1.89 (1.07–3.36) |  |
|  |  |  |  |  |
| Dilated pelvic | No | 39/332 (11.7) | 1 | 0.79 |
| vein(s) ^(##)^ | Yes | 5/48 (10.4) | 0.87 (0.33–2.34) |  |
|  |  |  |  |  |
| Dilated fallopian | No | 54/471 (11.5) | 1 | 0.90 |
| tube(s) | Yes | 2/16 (12.5) | 1.10 (0.24–4.99) |  |
|  |  |  |  |  |

(*) Odds ratio given for 10-unit increase in predictor variable. N=487 instead of 514 as 27 excluded due to amenorrhea. (#) N= 417 due to missing data. (+) Analysis excluding women with parity 0 (N= 167). (##) N= 380 due to missing data. (**) Unable to calculate odds ratio as no subjects in one category had moderate/severe menstrual hematochezia. Analysis using Fisher’s exact test. CI, confidence interval. BMI, body mass index. IBS, irritable bowel syndrome. IBD, inflammatory bowel disease.

**Table S14** Univariable analysis of demographic and clinical factors associated with EQ-VAS scores

| Variable | Category | EQ-VAS  Mean ± SD | Coefficient  (95% CI) | P-value |
| --- | --- | --- | --- | --- |
|  |  |  |  |  |
| Endometriosis | No | 69.7 ± 19.9 | 0 | 0.17 |
|  | Yes | 67.1 ± 20.1 | -2.7 (-6.2–1.2) |  |
|  |  |  |  |  |
| Age ^(*)^ | - | - | 0.1 (-2.1–2.3) | 0.91 |
|  |  |  |  |  |
| Ethnicity | White | 69.3 ± 19.0 | 0 | 0.88 |
|  | Black | 69.4 ± 19.9 | 0.1 (-5.5–5.7) |  |
|  | Asian | 67.0 ± 21.7 | -2.3 (-8.0–3.4) |  |
|  | Mixed / Other | 68.9 ± 21.5 | -0.4 (-4.7–3.9) |  |
|  |  |  |  |  |
| BMI (kg/m^2^) ^(*)^ | - | - | -3.8 (-6.7– -0.9) | **0.01** |
|  |  |  |  |  |
| BMI category ^(#)^ | Normal | 71.2 ± 19.1 | 0 | **0.03** |
|  | Underweight | 67.8 ± 15.6 | -3.5 (-12.9–6.0) |  |
|  | Pre-obesity | 70.3 ± 19.5 | -1.0 (-5.6–3.7) |  |
|  | Obesity class 1 | 63.9 ± 21.0 | -7.4 (-13.4– -1.3) |  |
|  | Obesity class 2/3 | 62.5 ± 22.6 | -8.7 (-15.6– -1.8) |  |
|  |  |  |  |  |
| Smoking status ^(##)^ | Non-smoker | 69.8 ± 20.4 | 0 | **0.04** |
|  | Ex-smoker | 70.4 ± 19.4 | 0.5 (-3.6–4.7) |  |
|  | Current smoker | 63.5 ± 18.6 | -6.3 (-11.4– -1.2) |  |
|  |  |  |  |  |
| Regular periods ^(+)^ | No | 69.4 ± 19.3 | 0 | 0.82 |
|  | Yes | 68.9 ± 20.3 | -0.5 (-4.5–3.6) |  |
|  |  |  |  |  |
| Period frequency | Normal | 68.7 ± 20.2 | 0 | 0.98 |
|  | Infrequent | 70.5 ± 20.0 | 1.8 (-6.6–10.1) |  |
|  | Frequent | 71.1 ± 24.9 | 2.4 (-8.0–12.8) |  |
|  | Variable | 69.4 ± 19.0 | 0.7 (-3.4–4.8) |  |
|  | No periods | 68.0 ± 20.5 | -0.7 (-8.6–7.2) |  |
|  |  |  |  |  |
| Period length ^(+)^ | Normal | 69.7 ± 20.0 | 0 | 0.25 |
|  | Prolonged | 63.5 ± 21.6 | -6.2 (-14.8–2.4) |  |
|  | Variable | 67.0 ± 19.5 | -2.7 (-8.0–2.6) |  |
|  |  |  |  |  |
| Gravidity | 0 | 71.1 ± 18.7 | 0 | **0.03** |
|  | 1 | 68.4 ± 18.9 | -2.7 (-7.2–1.8) |  |
|  | 2+ | 65.8 ± 22.4 | -5.3 (-9.4– -1.3) |  |
| Parity | 0 | 69.7 ± 18.9 | 0 | 0.14 |
|  | 1 | 70.7 ± 21.6 | 1.1 (-4.1–6.3) |  |
|  | 2+ | 65.6 ± 22.2 | -4.1 (-8.4–0.3) |  |
|  |  |  |  |  |
| Prior Cesarean | 0 | 67.1 ± 21.5 | 0 | 0.89 |
| delivery ^(++)^ | 1 | 68.2 ± 25.4 | 1.1 (-7.3–9.4) |  |
|  | 2+ | 69.3 ± 20.0 | 2.2 (-7.6–12.0) |  |
|  |  |  |  |  |
| Prior vaginal | 0 | 72.0 ± 20.7 | 0 | 0.29 |
| delivery ^(++)^ | 1 | 66.6 ± 24.4 | -5.4 (-14.2–3.4) |  |
|  | 2+ | 65.8 ± 21.0 | -6.3 (-14.4–1.9) |  |
|  |  |  |  |  |
| Analgesia use | No | 75.1 ± 17.3 | 0 | **0.002** |
|  | Yes | 67.8 ± 20.3 | -7.3 (-12.0– -2.6) |  |
|  |  |  |  |  |
| Hormonal | No | 68.9 ± 20.0 | 0 | 0.82 |
| contraception | Yes | 69.3 ± 20.2 | 0.5 (-3.6–4.6) |  |
|  |  |  |  |  |
| IBS | No | 69.4 ± 20.1 | 0 | **0.04** |
|  | Yes | 60.5 ± 16.4 | -8.9 (-17.4– -0.4) |  |
|  |  |  |  |  |
| IBD | No | 69.0 ± 20.0 | 0 | 0.92 |
|  | Yes | 70.0 ± 23.5 | 1.0 (-18.7–20.8) |  |
|  |  |  |  |  |
| Anxiety / depression | No | 69.5 ± 19.7 | 0 | **0.03** |
|  | Yes | 61.8 ± 22.8 | -7.7 (-14.7– -0.6) |  |
|  |  |  |  |  |
| Fibromyalgia | No | 69.4 ± 19.6 | 0 | **<0.001** |
|  | Yes | 46.9 ± 26.3 | -22.5 (-34.9– -10.1) |  |
|  |  |  |  |  |
| Migraine | No | 69.2 ± 19.8 | 0 | 0.29 |
|  | Yes | 64.9 ± 22.9 | -4.3 (-12.2–3.6) |  |
|  |  |  |  |  |
| Adenomyosis | No | 70.0 ± 20.3 | 0 | 0.06 |
|  | Yes | 66.4 ± 19.0 | -3.7 (-7.5–0.1) |  |
|  |  |  |  |  |
| Uterine fibroid(s) | No | 68.1 ± 19.9 | 0 | 0.16 |
|  | Yes | 70.7 ± 20.2 | 2.6 (-1.1–6.3) |  |
|  |  |  |  |  |
| Non-endometriotic | No | 69.5 ± 19.8 | 0 | 0.19 |
| ovarian cyst(s) | Yes | 66.4 ± 21.1 | -3.1 (-7.7–1.5) |  |
|  |  |  |  |  |
| Pelvic adhesions | No | 70.1 ± 19.5 | 0 | **0.04** |
|  | Yes | 66.1 ± 21.1 | -4.0 (-7.8– -0.1) |  |
|  |  |  |  |  |
| Dilated pelvic | No | 69.9 ± 19.9 | 0 | 0.37 |
| vein(s) ^(###)^ | Yes | 67.2 ± 20.0 | -2.7 (-8.7–3.3) |  |
|  |  |  |  |  |
| Dilated fallopian | No | 68.9 ± 20.0 | 0 | 0.85 |
| tube(s) | Yes | 69.9 ± 21.3 | 1.0 (-9.0–11.0) |  |
|  |  |  |  |  |

(*) Odds ratio given for 10-unit increase in predictor variable. (#) N= 438 due to missing data. (##) N=511 due to missing data. (+) Women with no periods excluded (N= 487). (++) Analysis excluding women with parity 0 (N= 175). (###) N= 403 due to missing data. SD, standard deviation. CI, confidence interval. BMI, body mass index. IBS, irritable bowel syndrome. IBD, inflammatory bowel disease.

**Table S15** Univariable analysis of demographic and clinical factors associated with EQ-5D scores

| Variable | Category | EQ-5D  Mean ± SD | Coefficient  (95% CI) | P-value |
| --- | --- | --- | --- | --- |
|  |  |  |  |  |
| Endometriosis | No | 0.72 ± 0.28 | 0 | 0.09 |
|  | Yes | 0.67 ± 0.33 | -0.05 (-0.11–0.01) |  |
|  |  |  |  |  |
| Age ^(*)^ | - | - | 0.00 (-0.03–0.04) | 0.82 |
|  |  |  |  |  |
| Ethnicity | White | 0.72 ± 0.28 | 0 | 0.85 |
|  | Black | 0.70 ± 0.27 | -0.02 (-0.06–0.06) |  |
|  | Asian | 0.68 ± 0.33 | -0.04 (-0.11–0.05) |  |
|  | Mixed / Other | 0.71 ± 0.31 | -0.01 (-0.07–0.06) |  |
|  |  |  |  |  |
| BMI (kg/m^2^) ^(*)^ | - | - | -0.09 (-0.13– -0.05) | **<0.001** |
|  |  |  |  |  |
| BMI category ^(#)^ | Normal | 0.75 ± 0.26 | 0 | **<0.001** |
|  | Underweight | 0.65 ± 0.32 | -0.09 (-0.23–0.04) |  |
|  | Pre-obesity | 0.75 ± 0.26 | 0.00 (-0.60–0.07) |  |
|  | Obesity class 1 | 0.60 ± 0.31 | -0.15 (-0.23– -0.06) |  |
|  | Obesity class 2/3 | 0.57 ± 0.40 | -0.17 (-0.28– -0.08) |  |
|  |  |  |  |  |
| Smoking status ^(##)^ | Non-smoker | 0.73 ± 0.27 | 0 | 0.12 |
|  | Ex-smoker | 0.69 ± 0.33 | -0.04 (-0.10–0.02) |  |
|  | Current smoker | 0.66 ± 0.32 | -0.07 (-0.15–0.00) |  |
|  |  |  |  |  |
| Regular periods ^(+)^ | No | 0.69 ± 0.31 | 0 | 0.47 |
|  | Yes | 0.71 ± 0.29 | 0.02 (-0.04–0.08) |  |
|  |  |  |  |  |
| Period frequency | Normal | 0.72 ± 0.28 | 0 | 0.77 |
|  | Infrequent | 0.70 ± 0.28 | -0.02 (-0.15–0.10) |  |
|  | Frequent | 0.68 ± 0.40 | -0.04 (-0.20–0.11) |  |
|  | Variable | 0.68 ± 0.32 | -0.04 (-0.10–0.02) |  |
|  | No periods | 0.73 ± 0.27 | 0.01 (-0.10–0.12) |  |
|  |  |  |  |  |
| Period length ^(+)^ | Normal | 0.72 ± 0.29 | 0 | 0.33 |
|  | Prolonged | 0.70 ± 0.28 | -0.02 (-0.14–0.11) |  |
|  | Variable | 0.66 ± 0.36 | -0.06 (-0.13–0.02) |  |
|  |  |  |  |  |
| Gravidity | 0 | 0.73 ± 0.27 | 0 | 0.19 |
|  | 1 | 0.72 ± 0.28 | -0.01 (-0.08–0.06) |  |
|  | 2+ | 0.67 ± 0.33 | -0.05 (-0.11–0.00) |  |
|  |  |  |  |  |
| Parity | 0 | 0.72 ± 0.28 | 0 | 0.30 |
|  | 1 | 0.72 ± 0.30 | 0.00 (-0.08–0.07) |  |
|  | 2+ | 0.67 ± 0.34 | -0.05 (-0.11–0.01) |  |
|  |  |  |  |  |
| Prior Cesarean | 0 | 0.68 ± 0.34 | 0 | 0.66 |
| delivery ^(++)^ | 1 | 0.73 ± 0.27 | 0.05 (-0.07–0.17) |  |
|  | 2+ | 0.67 ± 0.31 | 0.00 (-0.15–0.14) |  |
|  |  |  |  |  |
| Prior vaginal | 0 | 0.72 ± 0.26 | 0 | 0.79 |
| delivery ^(++)^ | 1 | 0.68 ± 0.35 | -0.04 (-0.16–0.09) |  |
|  | 2+ | 0.68 ± 0.33 | -0.04 (-0.16–0.08) |  |
|  |  |  |  |  |
| Analgesia use | No | 0.82 ± 0.22 | 0 | **<0.001** |
|  | Yes | 0.69 ± 0.30 | -0.13 (-0.20– -0.06) |  |
|  |  |  |  |  |
| Hormonal | No | 0.71 ± 0.30 | 0 | 0.64 |
| contraception | Yes | 0.70 ± 0.29 | -0.01 (-0.07–0.05) |  |
|  |  |  |  |  |
| IBS | No | 0.71 ± 0.29 | 0 | **0.03** |
|  | Yes | 0.57 ± 0.33 | -0.14 (-0.27– -0.02) |  |
|  |  |  |  |  |
| IBD | No | 0.71 ± 0.29 | 0 | 0.18 |
|  | Yes | 0.51 ± 0.47 | -0.20 (-0.49–0.09) |  |
|  |  |  |  |  |
| Anxiety / depression | No | 0.72 ± 0.29 | 0 | **<0.001** |
|  | Yes | 0.53 ± 0.37 | -0.19 (-0.29– -0.08) |  |
|  |  |  |  |  |
| Fibromyalgia | No | 0.72 ± 0.29 | 0 | **<0.001** |
|  | Yes | 0.33 ± 0.41 | -0.39 (-0.57– -0.21) |  |
|  |  |  |  |  |
| Migraine | No | 0.71 ± 0.29 | 0 | 0.55 |
|  | Yes | 0.67 ± 0.31 | -0.04 (-0.15–0.08) |  |
|  |  |  |  |  |
| Adenomyosis | No | 0.72 ± 0.29 | 0 | 0.15 |
|  | Yes | 0.68 ± 0.30 | -0.04 (-0.10–0.01) |  |
|  |  |  |  |  |
| Uterine fibroid(s) | No | 0.71 ± 0.29 | 0 | 0.71 |
|  | Yes | 0.70 ± 0.31 | -0.01 (-0.06–0.04) |  |
|  |  |  |  |  |
| Non-endometriotic | No | 0.71 ± 0.30 | 0 | 0.77 |
| ovarian cyst(s) | Yes | 0.72 ± 0.27 | 0.01 (-0.06–0.08) |  |
|  |  |  |  |  |
| Pelvic adhesions | No | 0.72 ± 0.29 | 0 | 0.14 |
|  | Yes | 0.68 ± 0.31 | -0.04 (-0.10–0.01) |  |
|  |  |  |  |  |
| Dilated pelvic | No | 0.71 ± 0.29 | 0 | 0.64 |
| vein(s) ^(###)^ | Yes | 0.73 ± 0.25 | 0.02 (-0.07–0.11) |  |
|  |  |  |  |  |
| Dilated fallopian | No | 0.71 ± 0.30 | 0 | 0.34 |
| tube(s) | Yes | 0.78 ± 0.20 | 0.07 (-0.08–0.22) |  |
|  |  |  |  |  |

(*) Odds ratio given for 10-unit increase in predictor variable. (#) N= 438 due to missing data. (##) N=511 due to missing data. (+) Women with no periods excluded (N= 487). (++) Analysis excluding women with parity 0 (N= 175). (###) N= 403 due to missing data. SD, standard deviation. CI, confidence interval. BMI, body mass index. IBS, irritable bowel syndrome. IBD, inflammatory bowel disease.

**Table S16** Multivariable analysis of factors associated with moderate-to-severe premenstrual pain

| Variable | Category | Odds Ratio (95% CI) | P-value |
| --- | --- | --- | --- |
|  |  |  |  |
| BMI (kg/m^2^) ^(*)^ | - | 1.55 (1.05–2.28) | 0.003 |
|  |  |  |  |
| Regular periods | No | 1 | 0.001 |
|  | Yes | 0.40 (0.23–0.68) |  |
|  |  |  |  |
| Analgesia use | No | 1 | 0.001 |
|  | Yes | 2.72 (1.53–4.80) |  |
|  |  |  |  |
| IBS | No | 1 | 0.03 |
|  | Yes | 4.84 (1.08–21.7) |  |
|  |  |  |  |
| Pelvic adhesions | No | 1 | 0.002 |
|  | Yes | 2.24 (1.34–3.73) |  |
|  |  |  |  |

(*) Odds ratio given for 10-unit increase in predictor variable. CI, confidence interval. BMI, body mass index. IBS, irritable bowel syndrome.

**Table S17** Multivariable analysis of factors associated with moderate-to-severe menstrual pain (dysmenorrhea)

| Variable | Category | Odds Ratio (95% CI) | P-value |
| --- | --- | --- | --- |
|  |  |  |  |
| Endometriosis | No | 1 | 0.09 |
|  | Yes | 1.72 (0.92–3.20) |  |
|  |  |  |  |
| Age ^(*)^ | - | 0.65 (0.46–0.92) | 0.02 |
|  |  |  |  |
| BMI (kg/m^2^) ^(*)^ | - | 1.88 (1.12–3.14) | 0.02 |
|  |  |  |  |
| Smoking | Non-smoker | 1 | 0.04 |
|  | Ex-smoker | 0.99 (0.55–1.79) |  |
|  | Current smoker | 6.78 (1.56–29.4) |  |
|  |  |  |  |
| Analgesia use | No | 1 | <0.001 |
|  | Yes | 3.67 (1.99–6.75) |  |
|  |  |  |  |

(*) Odds ratio given for 10-unit increase in predictor variable. CI, confidence interval. BMI, body mass index.

**Table S18** Multivariable analysis of factors associated with moderate-to-severe non-cyclical pain

| Variable | Category | Odds Ratio (95% CI) | P-value |
| --- | --- | --- | --- |
|  |  |  |  |
| Regular periods | No | 1 | 0.04 ^(+)^ |
|  | Yes | 0.66 (0.43–0.99) |  |
|  | No periods | 0.89 (0.38–2.08) |  |
|  |  |  |  |
| Analgesia use | No | 1 | 0.001 |
|  | Yes | 2.52 (1.48–4.31) |  |
|  |  |  |  |
| IBS | No | 1 | 0.02 |
|  | Yes | 3.11 (1.23–7.84) |  |
|  |  |  |  |
| Non endometriotic cyst(s) | No | 1 | 0.04 |
|  | Yes | 0.60 (0.37–0.99) |  |
|  |  |  |  |

(+) P-value for comparison of women with and without regular periods only (excluding amenorrheic women). CI, confidence interval. IBS, irritable bowel syndrome.

**Table S19** Multivariable analysis of factors associated with moderate-to-severe menstrual dyschezia

| Variable | Category | Odds Ratio (95% CI) | P-value |
| --- | --- | --- | --- |
|  |  |  |  |
| Endometriosis | No | 1 | <0.001 |
|  | Yes | 2.44 (1.59–3.78) |  |
|  |  |  |  |
| Age ^(*)^ | - | 0.67 (0.51–0.87) | 0.003 |
|  |  |  |  |
| Period length | Normal | 1 | 0.01 |
|  | Prolonged | 1.44 (0.55–3.74) |  |
|  | Variable | 0.32 (0.15–0.70) |  |
|  |  |  |  |
| Analgesia use | No | 1 | 0.06 |
|  | Yes | 1.79 (0.98–3.26) |  |
|  |  |  |  |
| IBS | No | 1 | 0.08 |
|  | Yes | 2.36 (0.91–6.10) |  |
|  |  |  |  |
| Fibromyalgia | No | 1 | 0.004 |
|  | Yes | 13.4 (2.31–77.5) |  |
|  |  |  |  |
| Migraine | No | 1 | 0.04 |
|  | Yes | 2.51 (1.02–6.17) |  |
|  |  |  |  |
| Non endometriotic cyst(s) | No | 1 | 0.02 |
|  | Yes | 0.48 (0.27–0.87) |  |
|  |  |  |  |

(*) Odds ratio given for 10-year increase in age. CI, confidence interval. IBS, irritable bowel syndrome.

**Table S20** Multivariable analysis of factors associated with moderate-to-severe non-menstrual dyschezia

| Variable | Category | Odds Ratio (95% CI) | P-value |
| --- | --- | --- | --- |
|  |  |  |  |
| Period frequency | Normal | 1 | 0.02 |
|  | Infrequent | 2.67 (1.06–6.77) |  |
|  | Frequent | 0.30 (0.04–2.38) |  |
|  | Variable | 1.01 (0.60–1.71) |  |
|  | No periods | 2.90 (1.22–6.88) |  |
|  |  |  |  |
| Parity | 0 | 1 | 0.06 |
|  | 1 | 0.43 (0.19–0.99) |  |
|  | 2+ | 1.32 (0.76–2.29) |  |
|  |  |  |  |
| Analgesia use | No | 1 | 0.04 |
|  | Yes | 2.23 (1.05–4.73) |  |
|  |  |  |  |
| IBD | No | 1 | 0.03 |
|  | Yes | 14.3 (1.32–1.56) |  |
|  |  |  |  |
| Fibromyalgia | No | 1 | 0.07 |
|  | Yes | 3.44 (0.87–13.4) |  |
|  |  |  |  |
| Anxiety / depression | No | 1 | 0.03 |
|  | Yes | 2.35 (1.07–5.14) |  |
|  |  |  |  |
| Pelvic adhesions | No | 1 | 0.03 |
|  | Yes | 1.73 (1.07–2.80) |  |
|  |  |  |  |

CI, confidence interval. IBD, inflammatory bowel disease.

**Table S21** Multivariable analysis of factors associated with moderate-to-severe lower back pain

| Variable | Category | Odds Ratio (95% CI) | P-value |
| --- | --- | --- | --- |
|  |  |  |  |
| BMI ^(*)^ | No | 2.16 (1.46–3.22) | <0.001 |
|  |  |  |  |
| Analgesia use | No | 1 | <0.001 |
|  | Yes | 4.23 (2.38–7.49) |  |
|  |  |  |  |
| IBS | No | 1 | 0.03 |
|  | Yes | 5.25 (1.14–24.2) |  |
|  |  |  |  |
| Uterine fibroid(s) | No | 1 | 0.002 |
|  | Yes | 0.50 (0.32–0.77) |  |
|  |  |  |  |
| Pelvic adhesions | No | 1 | 0.05 |
|  | Yes | 1.62 (0.99–2.63) |  |
|  |  |  |  |

(*) Odds ratio given for 10-unit increase in predictor variable. CI, confidence interval. BMI, body mass index. IBS, irritable bowel syndrome.

**Table S22** Multivariable analysis of factors associated with moderate-to-severe bladder pain

| Variable | Category | Odds Ratio (95% CI) | P-value |
| --- | --- | --- | --- |
|  |  |  |  |
| Analgesia | No | 1 | 0.04 |
|  | Yes | 2.29 (1.05–5.00) |  |
|  |  |  |  |
| Hormonal contraception | No | 1 | 0.008 |
|  | Yes | 1.97 (1.19–3.27) |  |
|  |  |  |  |
| Fibromyalgia | No | 1 | 0.005 |
|  | Yes | 7.83 (1.87–32.7) |  |
|  |  |  |  |
| Non endometriotic cyst(s) | No | 1 | 0.04 |
|  | Yes | 0.46 (0.22–0.96) |  |
|  |  |  |  |
| Pelvic adhesions | No | 1 | 0.007 |
|  | Yes | 1.95 (1.20–3.16) |  |
|  |  |  |  |

CI, confidence interval.

**Table S23** Multivariable analysis of factors associated with moderate-to-severe difficulty emptying bladder

| Variable | Category | Odds Ratio (95% CI) | P-value |
| --- | --- | --- | --- |
|  |  |  |  |
| Endometriosis | No | 1 | <0.001 |
|  | Yes | 2.56 (1.52–4.31) |  |
|  |  |  |  |
| Regular periods | No | 1 | 0.05 ^(+)^ |
|  | Yes | 2.04 (1.01–4.12) |  |
|  | No periods | 2.77 (0.85–9.06) |  |
|  |  |  |  |
| Fibromyalgia | No | 1 | 0.003 |
|  | Yes | 7.76 (2.01–29.9) |  |
|  |  |  |  |

(+) P-value for comparison between women with and without regular periods only (excluding amenorrheic women). CI, confidence interval.

**Table S24** Multivariable analysis of factors associated with moderate-to-severe urgency of bowel movements

| Variable | Category | Odds Ratio (95% CI) | P-value |
| --- | --- | --- | --- |
|  |  |  |  |
| BMI ^(*)^ | - | 1.51 (1.08–2.11) | 0.02 |
|  |  |  |  |
| Smoking | Non-smoker | 1 | 0.03 |
|  | Ex-smoker | 1.97 (1.21–3.21) |  |
|  | Current smoker | 1.19 (0.64–2.38) |  |
|  |  |  |  |
| Period frequency | Normal | 1 | 0.03 |
|  | Infrequent | 2.89 (1.12–7.45) |  |
|  | Frequent | 2.27 (0.68–7.59) |  |
|  | Variable | 0.80 (0.48–1.32) |  |
|  | No periods | 2.22 (0.85–5.79) |  |
|  |  |  |  |
| Analgesia use | No | 1 | 0.03 |
|  | Yes | 2.06 (1.06–3.98) |  |
|  |  |  |  |
| IBD | No | 1 | 0.04 |
|  | Yes | 10.5 (1.05–105) |  |
|  |  |  |  |
| Fibromyalgia | No | 1 | 0.007 |
|  | Yes | 20.0 (2.29–17.5) |  |
|  |  |  |  |

(*) Odds ratio given for 10 kg/m^2^ increase in BMI. CI, confidence interval. BMI, body mass index. IBD, inflammatory bowel disease.

**Table S25** Multivariable analysis of factors associated with moderate-to-severe incomplete bowel emptying

| Variable | Category | Odds Ratio (95% CI) | P-value |
| --- | --- | --- | --- |
|  |  |  |  |
| BMI (kg/m^2^) ^(*)^ | - | 1.60 (1.16–2.21) | 0.004 |
|  |  |  |  |
| Smoking | Non-smoker | 1 | 0.03 |
|  | Ex-smoker | 1.83 (1.14–2.94) |  |
|  | Current smoker | 1.63 (0.90–2.95) |  |
|  |  |  |  |

Odds ratio given for 10-kg/m2 increase in BMI. CI, confidence interval. (*). BMI, body mass index.

**Table S26** Multivariable analysis of factors associated with moderate-to-severe constipation

| Variable | Category | Odds Ratio (95% CI) | P-value |
| --- | --- | --- | --- |
|  |  |  |  |
| Analgesia use | No | 1 | 0.06 |
|  | Yes | 1.68 (0.98–2.89) |  |
|  |  |  |  |
| Anxiety / depression | No | 1 | <0.001 |
|  | Yes | 4.82 (2.20–10.6) |  |
|  |  |  |  |
| Migraine | No | 1 | 0.001 |
|  | Yes | 4.31 (1.80–10.3) |  |
|  |  |  |  |
| Non-endometriotic | No | 1 | 0.02 |
| ovarian cyst(s) | Yes | 0.54 (0.32–0.92) |  |
|  |  |  |  |
| Pelvic adhesions | No | 1 | 0.01 |
|  | Yes | 1.69 (1.12–2.55) |  |
|  |  |  |  |

CI, confidence interval.

**Table S27** Multivariable analysis of factors associated with moderate-to-severe menstrual hematochezia

| Variable | Category | Odds Ratio  (95% CI) | P-value |
| --- | --- | --- | --- |
|  |  |  |  |
| Fibromyalgia | No | 1 | 0.05 |
|  | Yes | 4.29 (1.03–17.9) |  |
|  |  |  |  |
| Pelvic adhesions | No | 1 | 0.03 |
|  | Yes | 1.93 (1.08–3.44) |  |
|  |  |  |  |

CI, confidence interval.

**Table S28** Multivariable analysis of factors associated with EQ-VAS scores

| Variable | Category | Coefficient (95% CI) | P-value |
| --- | --- | --- | --- |
|  |  |  |  |
| BMI ^(*)^ | - | -2.8 (-5.6–0.0) | 0.05 |
|  |  |  |  |
| Smoking | Non-smoker | 0 | 0.05 |
|  | Ex-smoker | 1.4 (-3.0–5.6) |  |
|  | Current smoker | -6.0 (-11.3– -0.6) |  |
|  |  |  |  |
| Analgesia use | No | 0 | 0.003 |
|  | Yes | -7.5 (-12.5– -2.6) |  |
|  |  |  |  |
| Anxiety / depression | No | 0 | 0.02 |
|  | Yes | -9.3 (-17.0– -1.6) |  |
|  |  |  |  |
| Fibromyalgia | No | 0 | <0.001 |
|  | Yes | -28.6 (-41.9– -15.2) |  |
|  |  |  |  |

(*) Regression coefficient given for 10 kg/m^2^ increase in BMI. CI, confidence interval. BMI, body mass index.

**Table S29** Multivariable analysis of factors associated with EQ-5D scores

| Variable | Category | Coefficient (95% CI) | P-value |
| --- | --- | --- | --- |
|  |  |  |  |
| Endometriosis | No | 0 | 0.06 |
|  | Yes | -0.05 (-0.11–0.00) |  |
|  |  |  |  |
| BMI (kg/m^2^) ^(*)^ | - | -0.08 (-0.12– -0.04) | 0.004 |
|  |  |  |  |
| Analgesia use | No | 0 | 0.001 |
|  | Yes | -0.12 (-0.19, –-0.05) |  |
|  |  |  |  |
| IBS | No | 0 | 0.007 |
|  | Yes | -0.17 (-0.30– -0.05) |  |
|  |  |  |  |
| IBD | No | 0 | 0.07 |
|  | Yes | -0.24 (-0.50–0.02) |  |
|  |  |  |  |
| Anxiety / depression | No | 0 | 0.05 |
|  | Yes | -0.11 (-0.22–0.00) |  |
|  |  |  |  |
| Fibromyalgia | No | 0 | <0.001 |
|  | Yes | -0.45 (-0.64– -0.27) |  |
|  |  |  |  |

(*) Regression coefficient given for 10-kg/m2 increase in BMI. CI, confidence interval. BMI, body mass index. IBS, irritable bowel syndrome. IBD, inflammatory bowel disease.
